# Insights on the Evolution of Mycoparasitism from the Genome of *Clonostachys rosea*

**DOI:** 10.1093/gbe/evu292

**Published:** 2015-01-08

**Authors:** Magnus Karlsson, Mikael Brandström Durling, Jaeyoung Choi, Chatchai Kosawang, Gerald Lackner, Georgios D. Tzelepis, Kristiina Nygren, Mukesh K. Dubey, Nathalie Kamou, Anthony Levasseur, Antonio Zapparata, Jinhui Wang, Daniel Buchvaldt Amby, Birgit Jensen, Sabrina Sarrocco, Emmanuel Panteris, Anastasia L. Lagopodi, Stefanie Pöggeler, Giovanni Vannacci, David B. Collinge, Dirk Hoffmeister, Bernard Henrissat, Yong-Hwan Lee, Dan Funck Jensen

**Affiliations:** ^1^Forest Mycology and Plant Pathology, Swedish University of Agricultural Sciences, Uppsala, Sweden; ^2^Department of Agricultural Biotechnology, Center for Fungal Pathogenesis, Seoul National University, Seoul, Korea; ^3^Department of Plant and Environmental Sciences and Copenhagen Plant Science Centre, University of Copenhagen, Copenhagen, Denmark; ^4^Department of Pharmaceutical Microbiology at the Hans-Knöll-Institute, Friedrich-Schiller-Universität, Jena, Germany; ^5^Department of Medical Biochemistry and Microbiology, Uppsala University, Uppsala, Sweden; ^6^Plant Pathology Laboratory, School of Agriculture, Aristotle University of Thessaloniki, Thessaloniki, Greece; ^7^INRA and Aix-Marseille Université, Polytech Marseille, UMR1163 Biotechnologie des Champignons Filamenteux, Marseille, France; ^8^Department of Agriculture, Food and Environment, University of Pisa, Pisa, Italy; ^9^Department of Agricultural Sciences, University of Helsinki, Helsinki, Finland; ^10^Department of Botany, School of Biology, Aristotle University of Thessaloniki, Thessaloniki, Greece; ^11^Department of Genetics of Eukaryotic Microorganisms, Institute of Microbiology and Genetics, Georg-August University, Göttingen, Germany; ^12^Centre National de la Recherche Scientifique (CNRS), UMR7257, Université Aix-Marseille, Marseille, France, and Department of Biological Sciences, King Abdulaziz University, Jeddah, Saudi Arabia.

**Keywords:** ABC transporter, biological control, *Bionectria ochroleuca*, fungicide, phylogeny, polyketide synthase

## Abstract

*Clonostachys rosea* is a mycoparasitic fungus that can control several important plant diseases. Here, we report on the genome sequencing of *C. rosea* and a comparative genome analysis, in order to resolve the phylogenetic placement of *C. rosea* and to study the evolution of mycoparasitism as a fungal lifestyle. The genome of *C. rosea* is estimated to 58.3 Mb, and contains 14,268 predicted genes. A phylogenomic analysis shows that *C. rosea* clusters as sister taxon to plant pathogenic *Fusarium* species, with mycoparasitic/saprotrophic *Trichoderma* species in an ancestral position. A comparative analysis of gene family evolution reveals several distinct differences between the included mycoparasites. *Clonostachys rosea* contains significantly more ATP-binding cassette (ABC) transporters, polyketide synthases, cytochrome P450 monooxygenases, pectin lyases, glucose-methanol-choline oxidoreductases, and lytic polysaccharide monooxygenases compared with other fungi in the Hypocreales. Interestingly, the increase of ABC transporter gene number in *C. rosea* is associated with phylogenetic subgroups B (multidrug resistance proteins) and G (pleiotropic drug resistance transporters), whereas an increase in subgroup C (multidrug resistance-associated proteins) is evident in *Trichoderma virens*. In contrast with mycoparasitic *Trichoderma* species, *C. rosea* contains very few chitinases. Expression of six group B and group G ABC transporter genes was induced in *C. rosea* during exposure to the *Fusarium* mycotoxin zearalenone, the fungicide Boscalid or metabolites from the biocontrol bacterium *Pseudomonas chlororaphis*. The data suggest that tolerance toward secondary metabolites is a prominent feature in the biology of *C. rosea*.

## Introduction

Agricultural chemicals for pest control and fertilization have played an important role for the substantial increase in the world’s food production during the past 50 years. The global human population is estimated to peak at around 9 billion people in the middle of this century, which will require further increases in food production. This must be achieved by producing more food from the same area of available land while reducing environmental impact (Godfray et al. 2010). Integrated pest management (IPM) is one approach to reduce the amount of pesticides used, through preventive cultural practices, the use of disease resistant plant cultivars, and the use of mechanical and biological control of pathogen populations. Biological control of plant pathogens by microbial antagonists is one promising component in future disease control strategies that is compatible with both organic agriculture and IPM.

One group of biological control agents (BCAs) includes mycotrophic fungi such as *Trichoderma* spp. and *Clonostachys* spp. (Druzhinina et al. 2011), which have the ability to parasitize and kill other fungi (mycoparasitism) and utilize dead fungal biomass (saprotrophy). These fungi can antagonize plant pathogenic fungi directly by secretion of cell wall degrading enzymes such as chitinases, β-1,3-glucanases, β-1,6-glucanases and proteases, and antibiotics such as peptaibols, gliotoxin, viridin and 6-pentyl-2*H*-pyran-2-one (Lorito et al. 2010; Druzhinina et al. 2011; Mukherjee et al. 2012), but also through competition for space and nutrients. Certain *Trichoderma* and *Clonostachys* species can also promote plant growth and elicit induced resistance that can protect plants from pathogen attack (Hermosa et al. 2012; Mukherjee et al. 2013). The exact mechanisms behind the disease control exerted by these BCAs may vary depending on the agricultural setting, including plant species, pathogen species, and environmental conditions. Therefore, a better understanding of the biological mechanisms that determine the outcome of biocontrol interactions is crucial for improving biological control in agricultural production systems.

*Clonostachys rosea* (Link: Fr.) Schroers, Samuels, Seifert & W. Gams, comb. nov. is the anamorph stage, and preferred name (Rossman et al. 2013), of the teleomorph *Bionectria ochroleuca* (Schw.) Schroers & Samuels (Schroers et al. 1999). *Clonostachys rosea* belongs to the order Hypocreales and the family Bionectriaceae, but its taxonomic position in relation with other families within Hypocreales is debated. James et al. (2006) reported Bionectriaceae to be sister taxa with the family Nectriaceae, with Hypocreaceae in a basal position, whereas Sung et al. (2007) reported Bionectriaceae as basal to both Nectriaceae and Hypocreaceae. The more studied mycotrophic *Trichoderma* spp. also belong to the order Hypocreales, but to the family Hypocreaceae. Hence, an understanding the phylogenetic relationships of Bionectriaceae, Nectriaceae, and Hypocreaceae within Hypocreales is a key for understanding evolution of mycotrophism as a fungal lifestyle. Comparative genomics of mycotrophic species from different families enables us to identify key similarities and differences between their respective life strategies, which has direct implications for the implementation of biocontrol in agriculture.

Recently, the sequenced and annotated genomes of the mycoparasitic species *Trichoderma atroviride*, *Trichoderma hamatum*, *Trichoderma longibrachiatum* and *Trichoderma virens*, and the saprotrophic *Trichoderma reesei*, were published (Martinez et al. 2008; Kubicek et al. 2011; Studholme et al. 2013; Xie et al. 2014). Comparative genomics revealed that the mycoparasitic lifestyle in *Trichoderma* was associated with gene copy number expansions of gene families involved in fungal cell wall degradation and secondary metabolite biosynthesis (Kubicek et al. 2011).

Here, we report the genome sequencing and analysis of the first species from the family Bionectriaceae; the mycoparasite *C. rosea*. A comparative analysis of gene family evolution is performed under the hypothesis that evolution of mycoparasitism in Bionectriaceae and Hypocreaceae results in selection for similar interaction mechanisms. However, the majority of expanded gene families in *C. rosea* does not evolve in the same manner in *Trichoderma* spp. *Clonostachys rosea* contain high numbers of polyketide synthases (PKSs) and ATP-binding cassette (ABC) transporters predicted to be involved in drug resistance, which emphasizes the role of secondary metabolites in *C. rosea* biology. We further show that several ABC transporter genes are induced by xenobiotic substances and illustrate the agro-industrial potential for *C. rosea* to be applied together with low dose fungicide treatments or other BCAs with complementing mode of action, to achieve additive disease control effects.

## Materials and Methods

A complete description of Materials and Methods is found in supplementary file S1, Supplementary Material online.

### Isolate and Culture Conditions

The *C. rosea* isolate IK726, originally isolated from barley roots in Denmark and evaluated extensively regarding its biocontrol efficiency (Jensen et al. 2007), was used for genome sequencing. The isolate was grown in potato dextrose broth (PDB; Thermo Scientific Oxoid, UK) for DNA extraction and on solid Vogel’s minimal medium with 1% (w/v) sucrose (Vogel 1956) for RNA extraction. DNA was extracted according to established methods (Sambrook and Russel 2001) and RNA was extracted using the RNeasy Plant Mini Kit (Qiagen, Hilden, Germany) according to manufacturer’s instructions.

### Genome Sequencing and Assembly

Base coverage of the *C. rosea* genome was generated using Illumina HiSeq paired end sequencing with an insert length of 0.5 kb and read length of 100 bp using standard library preparation kits. This datum was complemented with a mate pair library with 4 kb inserts sequenced on the Life Technologies SOLiD instrument with Exact Call Chemistry to generate sequence reads of 61 and 53 bp, forward and reverse, respectively. Illumina reads were quality trimmed using Nesoni clip (www.vicbioinformatics.com/software.nesoni, last accessed March 2014). The genome was then assembled with ABySS v. 1.3.3 (Simpson et al. 2009) with a k-mer length of 41.

### Gene Annotation

Protein-coding genes in the *C. rosea* genome were annotated using MAKER 2 (Holt and Yandell 2011). We configured MAKER 2 to use SNAP (Korf 2004), Augustus (Stanke et al. 2006), and GenemarkES (Ter-Hovhannisyan et al. 2008) for ab initio gene calls. Two RNA libraries of pure fungal culture were sequenced on the Illumina HiSeq, de novo assembled with Trinity (Grabherr et al. 2011) and then provided to MAKER 2 as expressed sequence tag evidence. The evidence set was also complemented with the predicted proteomes of *T. virens*, *T. atroviride*, *T. reseei,* and *Neurospora crassa*. Assembly sequences and annotation data for *C. rosea* IK726 is available from www.slu.se/Clonostachys-rosea-IK726 (last accessed January 13, 2015).

### Species Phylogeny

The program Composition Vector Tree (CVTree) version 4.2.1 (Xu and Hao 2009) was used for construction of a phylogenomic tree by using whole predicted proteome sequences. Ten species were included for the topology construction, including *C. rosea* (isolate IK726), *Fusarium graminearum* (isolate PH-1), *Fusarium oxysporum* forma specialis (f. sp.) *lycopersici* (isolate FOL 4287), *Fusarium solani* (isolate 77-13-4), *Fusarium verticillioides* (isolate 7600), *T. atroviride* (isolate IMI 206040), *T. reesei* (isolate QM6a), *T. virens* (isolate Gv29-8), *N. crassa* (isolate OR74A), and *Magnaporthe oryzae* (isolate 70-15). Except for *C. rosea*, the predicted proteome sequences of the included species were obtained from the Comparative Fungal Genomics Platform (Choi et al. 2013). A bootstrap test was performed by randomly resampling 63.21% of the proteomes for each species 100 times, as described previously (Zuo et al. 2010). The distance matrices generated by CVTree were converted into neighbor-joining trees by using “Neighbor” in the PHYLIP v. 3.6 package (Felsenstein 2005). A consensus tree of the resulting 100 CVTrees was calculated by using MEGA5 (Tamura et al. 2011) to evaluate bootstrap support of the topology.

### Gene Family Evolution

Branch lengths of the species topology generated by CVTree was calculated with PAML v. 4.4 (Yang 1997, 2007), based on a five-gene alignment including actin, calmodulin, glyceraldehyde 3-phosphate dehydrogenase, DNA-directed RNA polymerase II subunit B, and translation elongation factor 1 alpha. Coding gene sequences were retrieved from the respective genome sequences. Each gene was aligned individually using ClustalW (Larkin et al. 2007), and all alignments were then concatenated. The resulting alignment, containing all five genes, was used to calculate branch lengths using codeml in the PAML package (using a global model for d*N*/d*S*). The resulting species phylogeny was calibrated to the fossil record by setting the base of the Sordariomycetes to 335 Ma, according to the fossil recalibration by Lücking et al. (2009).

For manually annotated gene families, the predicted proteomes from individual fungal genome sequences were screened using BLASTP (Altschul et al. 1997) in an iterative process, using reference proteins that covered the diversity of the respective gene family (supplementary file S1, Supplementary Material online). OrthoMCL v. 2.0.8 (Li et al. 2003) was used to cluster protein sequences from the included fungal proteomes. Gene family evolution analysis was performed on families that contained ≥2 genes in at least one species and were present in ≥2 species. The program CAFE (Computational Analysis of gene Family Evolution) v. 3 (Han et al. 2013) was used to test whether gene family sizes were compatible with a stochastic birth and death model to estimate gene family size in extinct species and to identify lineages with accelerated rates of gene gain or loss. Mutation rate (λ) was estimated from the data and was 0.0024. A separate analysis on ABC transporter subgroups was performed and included data retrieved from *T. hamatum* (Studholme et al. 2013), *T. longibrachiatum* (Xie et al. 2014), and Kovalchuk and Driessen (2010). Branch lengths of the species topology were based on a five-gene alignment as described above, and λ was 0.001.

### Phylogenetic Analysis

ABC transporter amino acid sequences were aligned using ClustalW (Larkin et al. 2007) and phylogenetic analyses performed using Neighbor-Joining implemented in MEGA6 (Tamura et al. 2013). The JTT (Jones, Taylor, and Thorton) amino acid substitution model (Jones et al. 1992) was used with uniform rates among sites, and pairwise deletion of gaps. Statistical support for phylogenetic grouping was assessed by 100 bootstrap resamplings.

### Assessment of Fungicide and Metabolite Tolerance, and Biocontrol Efficiency

The in vitro effect of the fungicide Boscalid (BASF Hellas, Greece) on mycelial growth of *C. rosea* on PDA (Thermo Scientific Oxoid) was studied in triplicates in order to assess half maximal effective concentration (EC_50_). In dual culture assays, *Pseudomonas chlororaphis* strain MA342 (or sterile water in controls) was streaked at a 6-cm distance from an agar plug of *C. rosea* or *Microdochium nivale* (isolate MG1) in a 9-cm-diameter PDA or vegetative peptone agar (VPA, Thermo Scientific Oxoid) plate. Fungal growth was measured in triplicates as the distance between the inoculation point and the hyphal front after 8 days. Percentage fungal growth rate reduction by secreted *P. chlororaphis* metabolites was calculated as: (1 − [mean growth in *P. chlororaphis* plates/mean growth in control plates]) × 100.

Phenazine-1-carboxamide (PCN) was isolated from *P. chlororaphis* strain PCL1391 using Thin Layer Chromatography (TLC) as described previously (Keen et al. 1971). The TLC plate was covered with a thin layer of PDA containing 1 × 10^6^ *C. rosea* or *F. oxysporum* f. sp. *radicis lycopersici* conidia/ml, and assessment of growth on the PCN-containing area was done in triplicates. Biocontrol efficiency was evaluated using a gnotobiotic sand system (Simons et al. 1996), with minor modifications. Plant nutrient solution (Chin-A-Woeng et al. 1998) previously inoculated with 5 × 10^2^ *F. oxysporum* f. sp. *radicis lycopersici* conidia/ml was mixed with sand to give a final concentration of 50 conidia/g sand. Application of BCAs was done by dipping surface-sterilized tomato pregerminated seeds in a suspension of 1 × 10^7^ *P. chlororaphis* strain PCL1391 cells/ml, 1 × 10^6^ *C. rosea* conidia/ml, or both BCAs mixed together, prior to sowing into the sand. Ten days after sowing, assessment of disease severity was carried out in 20 replicates according to the following disease index scale: 1 = no visible symptoms, 2 = mild symptoms on roots, 3 = severe symptoms on roots and wilting, 4 = dead plants. Data were analyzed statistically by analysis of variance and compared using the Duncan test at *P* = 0.05, using the program SPSS version 16 (SPSS, IL).

Colonization of tomato roots by a green fluorescent protein (*gfp*)-expressing *C. rosea* IK726 isolate (Lübeck et al. 2002) was monitored using a Nikon D-Eclipse C1 confocal microscope, using the default filter set. Digital images were acquired with the manufacturer’s software. *Fusarium oxysporum* f. sp. *radicis lycopersici* was transformed to express red fluorescent protein (*rfp*) as described previously (Pantelides et al. 2009), and interactions with *gfp*-expressing *C. rosea* were studied in vitro (Bolwerk et al. 2003) and in planta (Lagopodi et al. 2002).

### Gene Expression Analysis

Gene expression of 11 ABC transporters (supplementary file S2, Supplementary Material online) was measured using quantitative reverse transcription polymerase chain reaction (RT-qPCR). Two *Fusarium* mycotoxins deoxynivalenol (DON) and zearalenone (ZEN), the fungicide Boscalid, and a 2 days postinoculation (dpi) culture filtrate of *P. chlororaphis* strain MA342 were used as exogenous toxic substances to generate environmental stresses with relevance for biocontrol conditions. *Clonostachys rosea* strain IK726 was cultured in 20 ml liquid Czapek-Dox media (Sigma-Aldrich, MO) in 250-ml Erlenmeyer flasks. After 3 dpi at 25 °C, the fungus was subjected to treatment with DON, ZEN, or Boscalid at a final concentration of 200 ppm. In the control treatment, DON and ZEN were replaced with an equal volume of methanol whereas Boscalid was replaced by water. For the *P. chlororaphis* culture filtrate treatment, *C. rosea* was cultured in 100 ml PDB for 7 dpi, harvested by filtration and placed in 100 ml *P. chlororaphis* culture filtrate or fresh VPB as control treatment. Fungal mycelia were subsequently collected after 2-h incubation by vacuum filtration. The harvested mycelia were frozen with liquid nitrogen and kept at −80 °C.

Total RNA was extracted from mycelia using RNeasy Plant Mini kit (Qiagen) according to the manufacturer’s protocol. To remove DNA impurities, treatment of DNase I and Ribolock RNAase inhibitor (Fermentas, Germany) was performed prior to cDNA synthesis using iScript cDNA synthesis kit (Bio-Rad, CA) following the manufacturer’s instructions. RT-qPCR was carried out as described previously (Kosawang et al. 2014), except that each treatment was measured in a minimum of three biological replicates, each based on two technical replicates. Gene expression data were analyzed by Student’s *t*-test with 95% confidential interval implemented in Statistica v.10 (StatSoft, OK).

## Results

### General Features of the *C. rosea* Genome

**Fig. 1.—:**
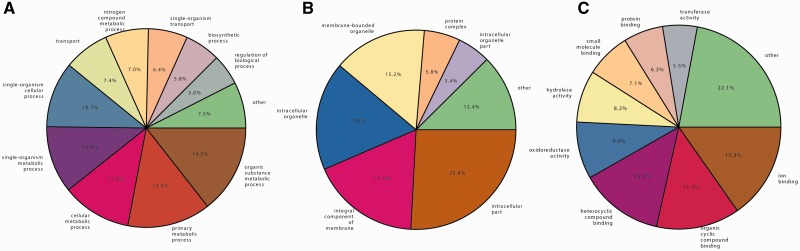
Functional annotations of *C. rosea* genes. GO terms of the three ontologies biological process (*A*), cellular component (*B*), and molecular function (*C*) were assigned using InterProScan 5.

The *C. rosea* genome (EMBL: PRJEB4200) was deep sequenced using a short read, whole-genome shotgun approach to at least 150× coverage and assembled with ABySS. The total assembly length was 58.3 Mb spread across 591 scaffolds longer than 2 kb (table 1). A total of 14,268 gene models coding for proteins were annotated using MAKER 2. RNA sequencing data as well as proteomes of related species were used for gene model prediction in MAKER 2. Descriptive names were assigned to 7,385 gene models based on homology to the Uniprot database where the best hit with *E* value below 1 × 10^−^^20^ was used. Finally, all gene models were functionally annotated using InterProScan5 to assign Gene Ontology (GO) terms (Ashburner et al. 2000) and Pfam domains (table 1, fig. 1). Pfam domains were found in 11,063 gene models, whereas GO terms were assigned to 8,918 genes.

**Table 1:** Statistics of the *Clonostachys rosea* Genome Assembly and Gene Annotations

	*C. rosea*
Genome size (Mb)	58.3
Number of scaffolds	595
Scaffold N50 (kb)	790
Gap length (kb)	924
Gene count	14,268
Total coding length (Mb)	21.0
Genes with EST support	7,854
Average/median transcript length (bp)	1,797.26/1,544
Average/median exon count	3.2/3

### Phylogenetic Placement of *C. rosea*

**Fig. 2.—:**
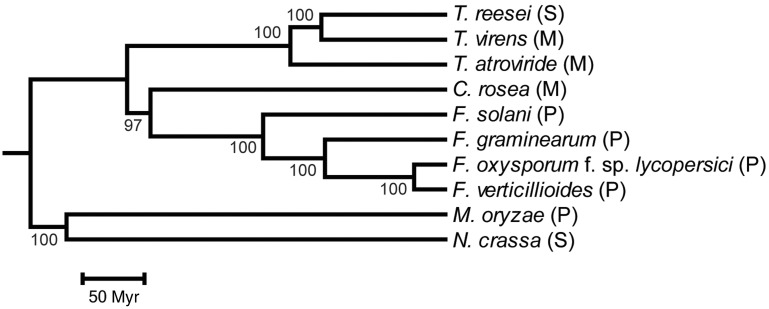
Phylogenetic placement of *C. rosea* within Hypocreales. A phylogenomic analysis was performed with the program CVTree on whole predicted proteomes of the included species. Bootstrap support values ≥97% are associated with lineages. Branch lengths of the species topology generated by CVTree was calculated with PAML, based on a five-gene alignment including actin, calmodulin, glyceraldehyde 3-phosphate dehydrogenase, DNA-directed RNA polymerase II subunit B, and translation elongation factor 1 alpha. The species phylogeny was calibrated to the fossil record by setting the base of the Sordariomycetes to 335 Ma. The included species represents the order Hypocreales: *C. rosea* (family Bionectriaceae), *F. graminearum*, *F. oxysporum* f. sp. *lycopersici*, *F. solani*, *F. verticillioides* (family Nectriaceae), *T. atroviride*, *T. reesei*, *T. virens* (family Hypocreaceae), the order Sordariales (*N. crassa*), and the order Magnaporthales (*M. oryzae*). M, mycoparasite; P, plant pathogen; S, saprotroph.

In order to resolve the phylogenetic relationship between Bionectriaceae, Nectriaceae, and Hypocreaceae within the order Hypocreales, a whole-proteome analysis was conducted with CVTree. The phylogenetic analysis included *C. rosea* (Bionectriaceae), *F. graminearum*, *F. oxysporum* f. sp. *lycopersici*, *F. solani* and *F. verticillioides* (Nectriaceae), *T. atroviride*, *T. reesei* and *T. virens* (Hypocreaceae) and the outgroup species *N. crassa* (order: Sordariales) and *M. oryzae* (order: Magnaporthales). Based on the whole-proteome analysis, *C. rosea* clustered as a sister taxa to *Fusarium* spp. with 97% bootstrap support, with *Trichoderma* spp. in a basal position (fig. 2). Among *Fusarium* spp., *F. oxysporum* f. sp. *lycopersici* and *F. verticillioides* clustered as sister taxa, whereas *F. solani* formed the most basal lineage. *Trichoderma reesei* and *T. virens* were identified as sister taxa, with *T. atroviride* in a basal position.

### The Mating Type Locus

As *C. rosea* is reported to be homothallic (Schroers et al. 1999), the structure of the mating type locus was analyzed. The genome of *C. rosea* contained two mating type (MAT) 1-2-1 orthologs (BN8691_T00013892_1 on scaffold 161 and BN8691_T000141771_1 on scaffold 347). The high sequence similarity of the genes (100%) and scaffolds (99%) suggests that the apparent duplication was an artifact due to the assembly. The predicted *mat1-2-1* gene encoded a predicted protein of 242 amino acids with a conserved high mobility group (HMG) domain, with high similarity (*E* value = 2 × 10^−^^77^) to the mating type 2 HMG-protein in *Epichloe festucae* (GenBank: AEI72619). No clear orthologs to the MAT1-1 proteins were found in *C. rosea*. The APN2 DNA lyase and the SLA2 cytoskeleton assembly control factor have been reported neighboring the MAT loci in many other ascomycetes (Debuchy et al. 2010). Both genes, *apn2* (BN8691_T00013667_1) and *sla2* (BN8691_T00013890_1) were predicted in *C. rosea*. The *sla2* gene was located on scaffold 161, upstream from *mat1-2-1*, and therefore we considered scaffold 161 to contain the MAT1-2 locus in *C. rosea*. The *apn2* gene was located in the end of scaffold 113 and may be adjacent to *mat1-2-1*, as *mat1-2-1* was at the end of scaffold 161. One additional gene (BN8691_T00013891_1) was predicted to be located between *sla2* and *mat1-2-1*, and displayed some sequence similarities with the MAT1-2-3 proteins of *Fusarium* species (Martin et al. 2011), but the highest similarity (*E* value = 3 × 10^−^^11^) was toward a hypothetical protein from *T. virens* (GenBank: EFY88586).

### Analysis of Gene Family Evolution

Gene family expansions and contractions in *C. rosea* were compared with nine other Sordariomycete genomes, with varying life strategies (fig. 2). Gene family data in extant species were either manually annotated (154 families), or predicted with OrthoMCL (17,138 families). The program CAFE was used to test whether gene family sizes were compatible with a stochastic birth and death model, for estimation of gene family size in extinct species and for identification of lineages with accelerated rates of gene gains or losses (supplementary file S3, Supplementary Material online).

In total, 91 gene families were identified to evolve nonrandomly (*P* ≤ 0.05), although changes in 62 families were restricted to *F. oxysporum* f. sp. *lycopersici*, *F. verticillioides*, and their ancestor (table 2). The genome of *C. rosea* contained significantly more polysaccharide lyase family 1 (PL1, pectin lyase), auxiliary activity family 3 (AA3, glucose-methanol-choline oxidoreductases), AA9 (lytic polysaccharide monooxygenase), ABC transporter, PKS, cytochrome P450 monooxygenase (CYP) and OrthoMCL family 1001 (PKS), OrthoMCL family 1003 (ABC transporter), and OrthoMCL family 1044 (Ankyrin-repeat protein) genes than predicted under a random model, but significantly fewer carbohydrate-binding family 18 (CBM18, chitin-binding) module-containing genes (table 2). This was in sharp contrast to *Trichoderma* spp. as no gene family identified as expanded in *C. rosea* was expanded in any *Trichoderma* species, with the exception for CYP that was expanded in *T. virens* and contracted in *T. atroviride* and *T. reesei* (table 2). When compared with *Fusarium* spp., AA3 was identified as expanded in *F. solani*, ABC transporter was expanded in *F. oxysporum* f. sp. *lycopersici*, whereas CYP was expanded in *F. oxysporum* f. sp. *lycopersici* and *F. solani*. The CBM18 and CYP gene families evolved nonrandomly in a majority of the ten included species (seven and ten species, respectively). *Fusarium oxysporum* f. sp. *lycopersici* contained the highest number of expanded gene families (79), whereas *F. verticillioides* contained the highest number of contracted gene families (22).

**Table 2:** Gene Numbers in Nonrandomly Evolving Gene Families

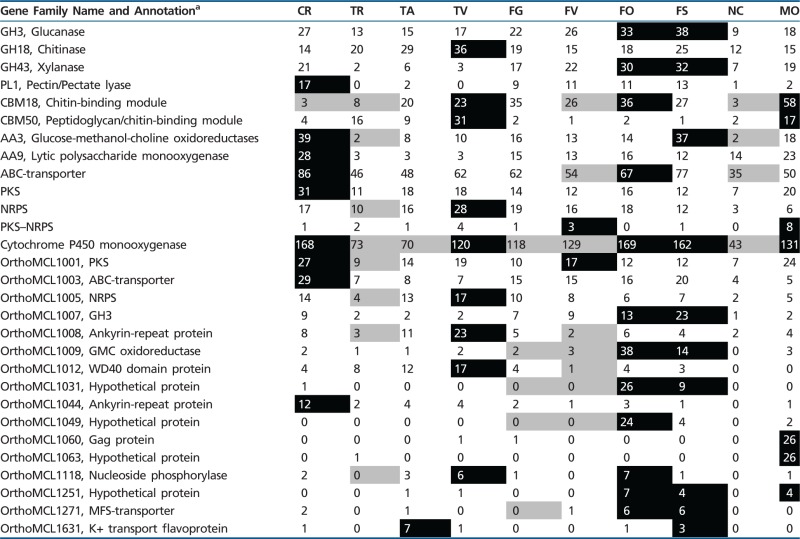

Note.—Gene numbers boxed in black indicate a significant (*P* ≤ 0.05) expansion, whereas gene numbers boxed in gray indicate a significant (*P* ≤ 0.05) contraction of gene family size. Note that 62 gene families with changes restricted to *Fusarium oxysporum* f. sp. *lycopersici*, *Fusarium verticillioides* and their ancestor is not shown (see supplementary file S3, Supplementary Material online).

^a^Annotation is based on BLASTP analysis (*E* value ≤ 1e-10) of the NCBI nr database, or from the SMART protein analysis tool. Species abbreviations: CR, *Clonostachys rosea*; FG, *Fusarium graminearum*; FO, *Fusarium oxysporum* f. sp. *lycopersici*; FS, *Fusarium solani*; FV, *Fusarium verticillioides*; MO, *Magnaporthe oryzae*; NC, *Neurospora crassa*; TA, *Trichoderma atroviride*; TR, *Trichoderma reesei*; TV, *Trichoderma virens*.

### Carbohydrate-Active Enzymes

The PL1 gene family, which was significantly expanded in *C. rosea* (17 genes, supplementary file S4, Supplementary Material online) compared with all other included species, contained enzymes with pectin degrading activity (pectate lyase, EC 4.2.2.2; exo-pectate lyase, EC 4.2.2.9; pectin lyase, EC 4.2.2.10) as listed in the CAZy (carbohydrate-active enzymes) database (Lombard et al. 2014). Furthermore, a more detailed analysis of the AA3 gene family was conducted, as this family consists of four defined subfamilies (AA3_1 through AA3_4; Levasseur et al. 2013). This analysis showed that the expansion in *C. rosea* and *F. solani* and the contraction in *T. reesei* and *N. crassa* took place in AA3_2 (supplementary file S4, Supplementary Material online). The AA3_2 subfamily included two closely related H_2_O_2_-producing enzyme classes, aryl-alcohol oxidase (AO, EC 1.1.3.7) and glucose 1-oxidase (GOX, EC 1.1.3.4, Levasseur et al. 2013). The significantly lower number of chitin-binding CBM18s, and the low number of peptidoglycan/chitin-binding CBM50s, in *C. rosea* was related to the low number of GH18 chitinases (EC 3.2.1.14) in this species. More specifically, CBM18s are found exclusively in GH18 subgroup C (killer toxin-like chitinases), but not in GH18 subgroup A (predicted to contain mostly exo-acting chitinases) or GH18 subgroup B (endo-acting chitinases) (Seidl 2008). A more detailed analysis confirmed that *C. rosea* only contained two B group and two C group GH18 chitinases, whereas the number of group A GH18s (eight genes) was comparable to the other included species (supplementary file S4, Supplementary Material online).

### Proteins Involved in Secondary Metabolite Biosynthesis

**Fig. 3.—:**
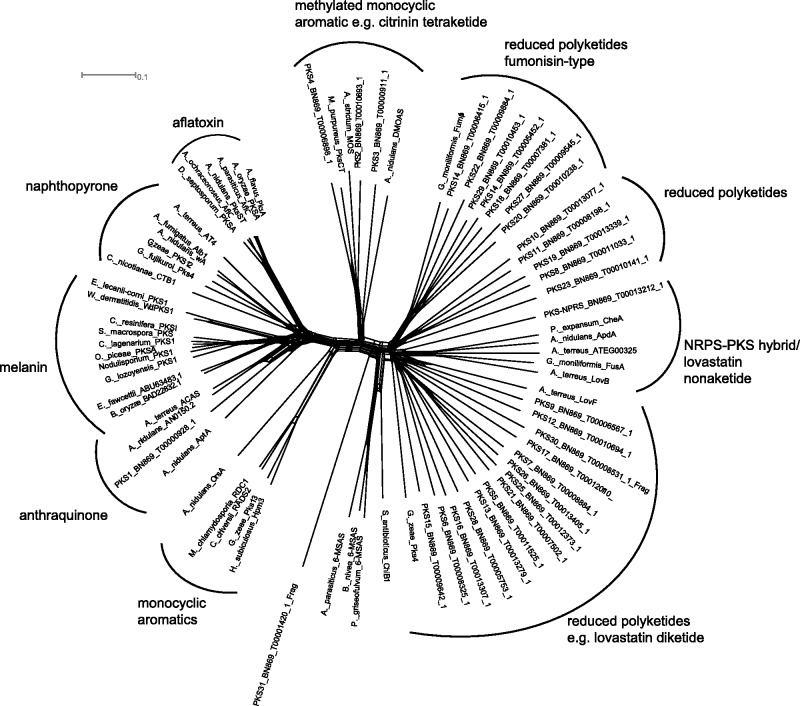
Phylogenetic network of *C. rosea* PKS genes. Ketosynthase domains were aligned with those of from experimentally characterized PKSs of ascomycetes and bacteria. The network was constructed using the neighbor-net method, implemented in the SplitsTree4 program. The scale bar indicates the uncorrected pairwise distance. HR-PKS = highly reducing PKS; NR-PKS = nonreducing PKS; PR-PKS = partially reducing PKS. Full species names and accession numbers are given in supplementary file S5, Supplementary Material online.

*Clonostachys rosea* harbored an impressive repertoire of genes coding for PKS and nonribosomal peptide synthetase (NRPS) secondary metabolite assembly lines, with 31 PKS, 17 NRPS, and 1 PKS–NRPS hybrid (supplementary file S5, Supplementary Material online). Furthermore, eight terpenoid synthase genes were identified. In order to assign the PKS genes to functional categories, a phylogenetic network comprising keto synthase domains of all deduced PKSs from *C. rosea*, combined with functionally characterized PKSs of other ascomycetes, was performed (fig. 3). This functional clustering revealed that most PKS genes code for highly reducing PKSs, whereas only four PKSs (PKS1–PKS4) clustered with nonreducing (aromatic) PKSs. The phylogenetic analysis placed PKS1 with anthraquinone-type PKSs, whereas PKS2 and PKS4 represented the citrinin-type of tetraketide synthases. PKS3 resembled a dimethyl orsellinic acid synthase.

The product spectrum of NRPSs encoded in the *C. rosea* genome was predicted by domain analysis and extraction of amino acid specificity codes (Stachelhaus et al. 1999) (supplementary file S5, Supplementary Material online). These results suggested that *C. rosea* possess one putative epipolythiodiketopiperazine synthetase (NPS1), which is presumably involved in biosynthesis of the antimicrobial compound glioperazine (Usami et al. 2004) or a related metabolite. Furthermore, several putative peptaibol assembly lines were identified. The largest peptaibol synthetase in *C. rosea* (NPS9) comprised 20 biosynthetic modules, whose specificity signatures corresponded well to the structure of antimicrobial peptaibols isolated from *C. rosea* (Rodriguez et al. 2011). However, the majority of NRPS genes belonged to orphan pathways.

### Membrane Transporters

*Clonostachys rosea* contained not only the highest number of predicted ABC transporters (86 genes, supplementary file S6, Supplementary Material online) among the included species but also the highest number of predicted major facilitator superfamily (MFS) transporters (620 genes, supplementary file S6, Supplementary Material online). The OrthoMCL analysis identified six MFS transporter genes in two families (OrthoMCL12606 and OrthoMCL12615) that were unique for *C. rosea* (supplementary file S7, Supplementary Material online).

**Fig. 4.—:**
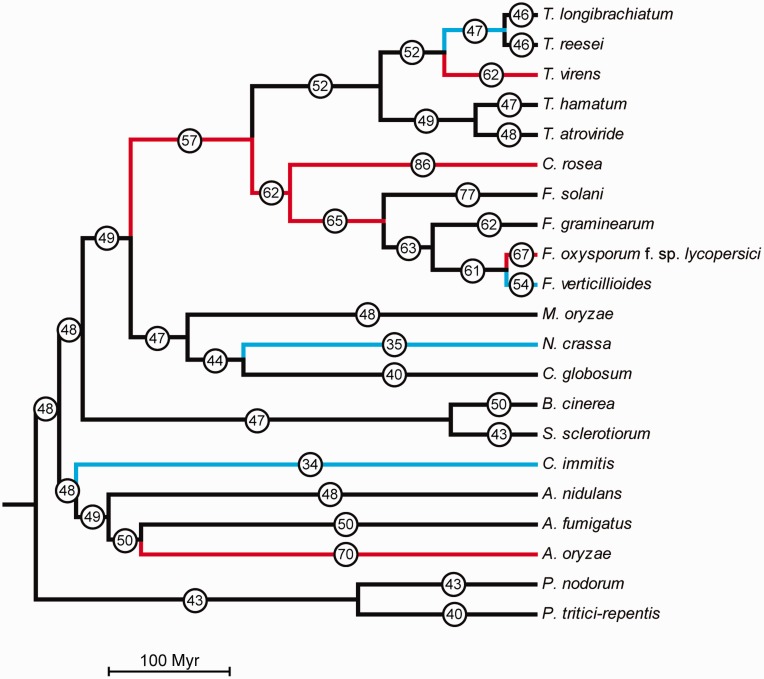
Distribution of ABC transporter gene gain and loss among fungal species. Phylogenetic relationships among the fungal species used in the current analysis are shown. Circled numbers represent total number of ABC transporter genes in extant species and estimates of total number of ABC transporter genes for ancestral species. Red lineages indicate a significant (*P* ≤ 0.05) expansion of ABC transporter genes, whereas blue lineages indicate significant (*P* ≤ 0.05) contractions of ABC transporter genes.

The ABC transporter gene family is shown to be divided into groups with various functions (Kovalchuk and Driessen 2010). As mycoparasites need to defend themselves against toxic metabolites produced by the fungal prey, we hypothesized that the increased number of ABC transporters in *C. rosea* was related to drug resistance. Therefore, a more detailed evolutionary analysis of ABC transporters in filamentous ascomycetes was performed. The analysis of gene family evolution estimated the number of ABC transporter genes in ancestral species (fig. 4) and number of gene gains and losses for each ABC transporter group in each branch of the species tree (supplementary file S6, Supplementary Material online).

**Fig. 5.—:**
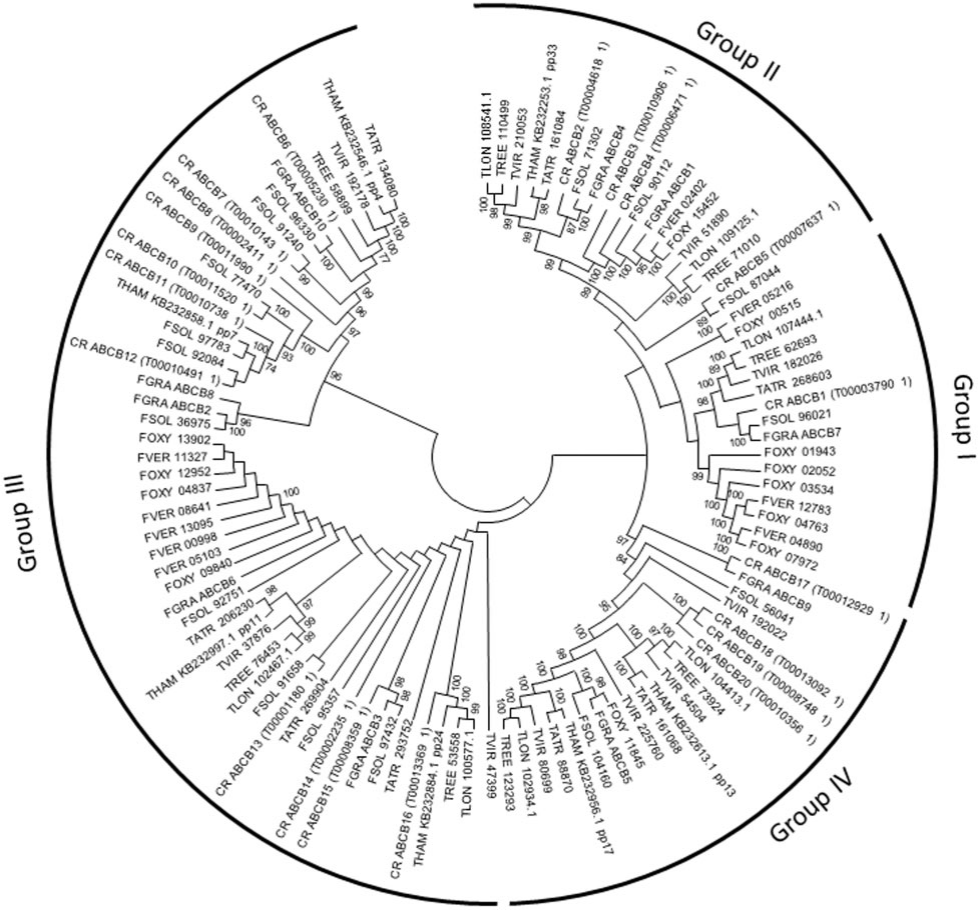
Phylogenetic relationships of ABC transporter subgroup B among Hypocreales. Predicted amino acid sequences of ABC transporters were aligned by ClustalW and used to construct a phylogenetic tree using the Neighbor-Joining method. ABC B subgroups were designated according to Kovalchuk and Driessen (2010). Analysis was performed with the MEGA6 software package. Bootstrap support (≥70%) values are associated with nodes. CR = *C. rosea*; FGRA = *F. graminearum*; FOXY = *F. oxysporum* f. sp. *lycopersici*; FSOL = *F. solani*; FVER = *F. verticillioides*; TATR = *T. atroviride*; THAM = *T. hamatum*; TLON = *T. longibrachiatum*; TREE = *T. reesei*; TVIR = *T. virens*.

**Fig. 6.—:**
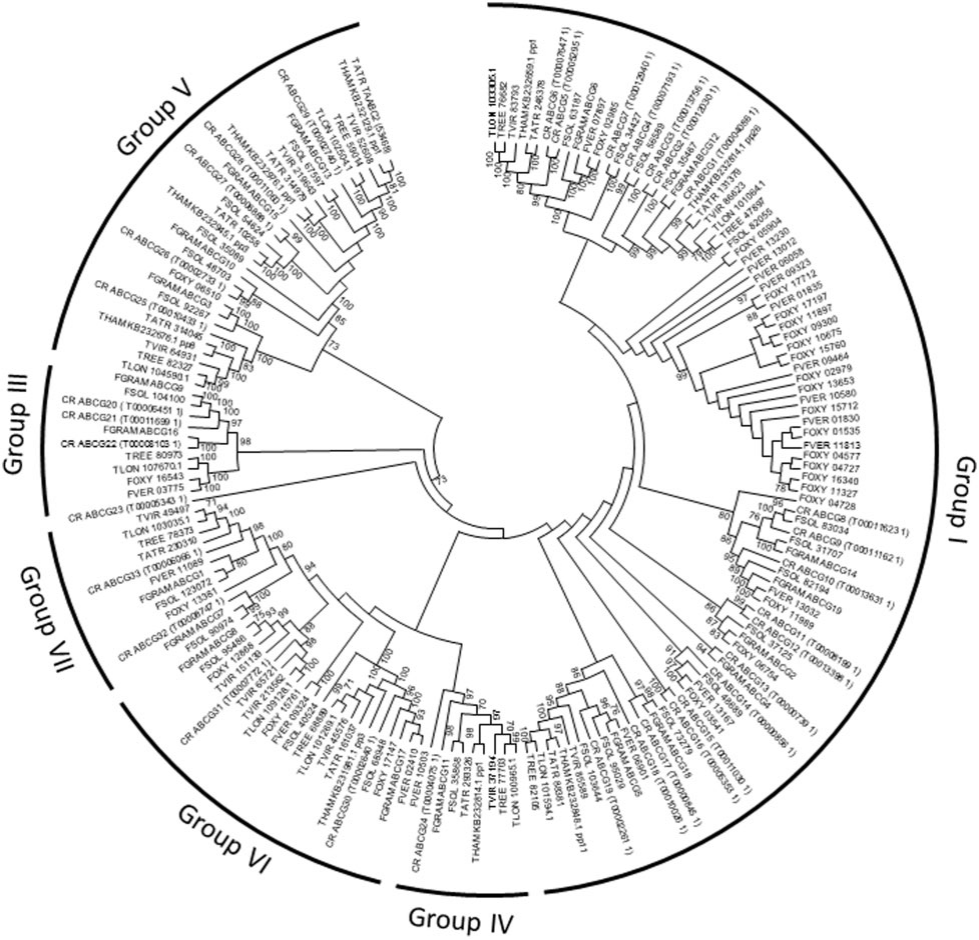
Phylogenetic relationships of ABC transporter subgroup G among Hypocreales. Predicted amino acid sequences of ABC transporters were aligned by ClustalW and used to construct a phylogenetic tree using the Neighbor-Joining method. ABC G subgroups were designated according to Kovalchuk and Driessen (2010). Analysis was performed with the MEGA6 software package. Bootstrap support (≥70%) values are associated with nodes. CR = *C. rosea*; FGRA = *F. graminearum*; FOXY = *F. oxysporum* f. sp. *lycopersici*; FSOL = *F. solani*; FVER = *F. verticillioides*; TATR = *T. atroviride*; THAM = *T. hamatum*; TLON = *T. longibrachiatum*; TREE = *T. reesei*; TVIR = *T. virens*.

The analyses showed that the expansion of ABC transporters in *C. rosea* took place in group B (multidrug resistance proteins) and in group G (pleiotropic drug resistance proteins) (*P* ≤ 0.001, table 3). ABC transporters in groups B and G are further divided into subgroups (Kovalchuk and Driessen 2010), and phylogenetic analyses showed that most group B ABC transporters in *C. rosea* were found in subgroup B-III (fig. 5), which include the *M. oryzae* B-III member Abc3 that is partly required for resistance toward valinomycin, actinomycin D, and reactive oxygen species (Sun et al. 2006). The expansion in group G involved subgroups G-I and G-V (fig. 6), which include many members with established roles in drug resistance (Ruocco et al. 2009; Kovalchuk and Driessen 2010).

**Table 3:** Nonrandomly Evolving Branches in the Fungal ABC Transporter Gene Family

Data Set	Branch ID	*P* value	Change^a^
All ABC transporters		0.003	
	Ancestor to Hypocreales	0.019	8
	Ancestor to *Trichoderma longibrachiatum/Trichoderma reesei*	0.030	−5
	*Trichoderma virens*	0.001	10
	Ancestor to Bionectriaceae/Nectriaceae	0.020	5
	*Clonostachys rosea*	<0.001	24
	Ancestor to *Fusaria*	0.006	3
	*Fusarium oxysporum* f. sp. *lycopersici*	0.003	6
	*Fusarium verticillioides*	<0.001	−7
	*Neurospora crassa*	0.054	−9
	*Coccidioides immitis*	0.023	−14
	*Aspergillus oryzae*	0.002	20
ABC-B		0.017	
	*C. rosea*	0.001	10
	*F. oxysporum* f. sp. *lycopersici*	0.001	4
	*F. verticillioides*	0.004	−3
	*A. oryzae*	0.057	6
ABC-C		0.010	
	*T. virens*	<0.001	7
	*F. oxysporum* f. sp. *lycopersici*	0.028	−2
	*C. immitis*	0.037	−6
	*A. oryzae*	0.017	7
ABC-G		≤0.001	
	Ancestor to *Trichoderma*	0.032	−4
	*T. reesei*	0.018	−2
	Ancestor to Bionectriaceae/Nectriaceae	0.017	3
	*C. rosea*	<0.001	14
	*Fusarium solani*	0.049	5
	*F. oxysporum* f. sp. *lycopersici*	<0.001	6
	*F. verticillioides*	0.009	−3
	*A. oryzae*	0.015	8

^a^Gene family size change as compared with the most recent ancestor.

In *Trichoderma*, a significant increase (*P* ≤ 0.001) of ABC transporter genes was identified for *T. virens*, which could be traced to group ABC C (multidrug resistance-associated proteins), subgroup C-V (supplementary file S8, Supplementary Material online) that contain members hypothesized to be involved in export of endogenous toxins (von Döhren 2009). In contrast, *T. reesei* and the ancestor to *Trichoderma* experienced a significant (*P* ≤ 0.032) loss of group G ABC transporters (table 3). Significant losses (*P* ≤ 0.054) of ABC transporter genes were also detected in *Coccidioides immitis*, *F. verticillioides* and *N. crassa*, whereas an expansion (*P* ≤ 0.003) was evident in *Aspergillus oryzae* and *F. oxysporum* f. sp. *lycopersici* (table 3).

### Gene Expression of ABC Transporters

The expansion of ABC transporters associated with drug resistance in *C. rosea* prompted us to investigate whether expression of ABC transporter genes was induced in response to mycotoxins DON and ZEN, the fungicide Boscalid and metabolites from the BCA bacterium *P. chlororaphis* strain MA342. First, the ability of *C. rosea* to tolerate Boscalid was evaluated by measuring in vitro growth on agar plates, and the resulting EC_50_ value was greater than 500 µg/ml. Second, the ability of *C. rosea* to tolerate metabolites produced by *P. chlororaphis* strain MA342 was evaluated using a dual culture assay. The growth inhibition of the snow mould pathogen *Mi. nivale* by secreted *P. chlororaphis* MA342 metabolites was significantly (*P* ≤ 0.05) higher than the growth reduction of *C. rosea* under the same conditions, on both PDA and VPA (supplementary file S9, Supplementary Material online).

**Fig. 7.—:**
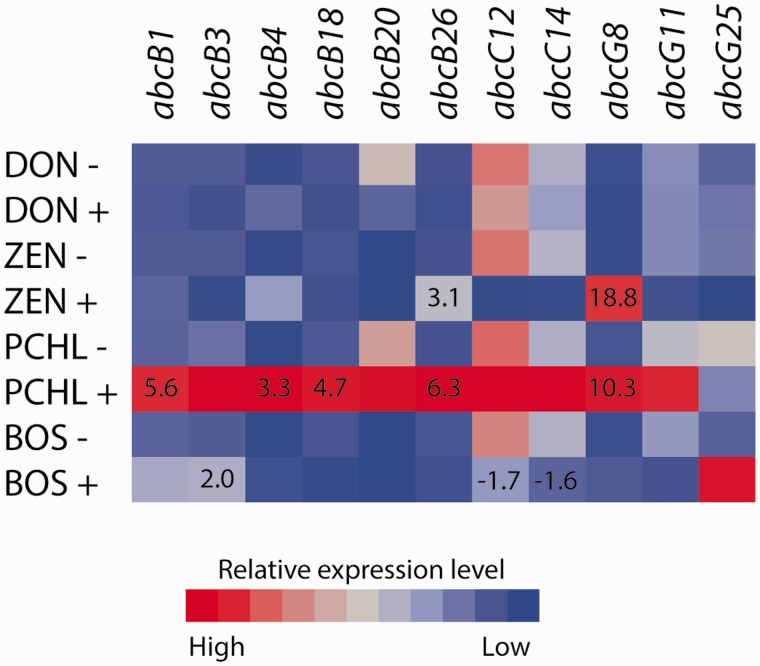
Gene expression of selected ABC transporters in *C. rosea*. Gene expression of ABC transporters was assayed by RT-qPCR during exposure of *C. rosea* to deoxynivalenone (DON+), zearalenone (ZEN+), Boscalid (BOS+) and *P. chlororaphis* metabolites (PCHL+), and compared with the corresponding control (−). Relative expression levels are color-coded. Statistically significant (*P* ≤ 0.05) changes are indicated by fold change numbers.

Expression of a subset of 11 genes representing groups ABC B, ABC C, and ABC G was analyzed (supplementary file S2, Supplementary Material online). Two ABC transporter genes were significantly (*P* ≤ 0.043) induced by the ZEN treatment; *abcB26* and *abcG8* (fig. 7). Gene *abcB3* was induced (*P* = 0.012) by the Boscalid treatment whereas two genes (*abcC12* and *abcC14*) were repressed (*P* ≤ 0.048) (fig. 7). Five genes were induced (*P* ≤ 0.037) by exposure to a culture filtrate from *P. chlororaphis*; *abcB1*, *abcB4*, *abcB18*, *abcB26,* and *abcG8* (fig. 7). No gene expression changes were induced by the DON treatment.

### Biological Control Assay

The high tolerance of *C. rosea* toward metabolites from *P. chlororaphis* suggested the potential for combination treatments of these two BCAs for additive biocontrol effects. For this experiment, *P. chlororaphis* strain PCL1391 was used. The ability of *C. rosea* to tolerate the main metabolite from PCL1391, PCN (Chin-A-Woeng et al. 1998), was evaluated. PCN completely inhibited in vitro growth of the tomato foot and root rot pathogen *F. oxysporum* f. sp. *radicis lycopersici*, whereas growth of *C. rosea* was observed under the same condition (supplementary file S9, Supplementary Material online). A gnotobiotic sand system was used to evaluate the effect of coinoculating *C. rosea* together with *P. chlororaphis* PCL1391 on tomato seeds to protect against *F. oxysporum* f. sp. *radicis lycopersici* foot and root rot disease. The combination treatment of the two BCAs reduced the disease index to a significantly (*P* ≤ 0.05) lower level than either the single *C. rosea* or the single *P. chlororaphis* treatments (supplementary file S9, Supplementary Material online).

Colonization of tomato seedling roots by *C. rosea* IK726 in the gnotobiotic sand system was studied by fluorescence microscopy using a strain expressing *gfp*. *Clonostachys rosea* colonized the plant’s roots by growing along the junctions of epidermal cells, formed a net of hyphae around the main root and conidiated 6 dpi (supplementary file S9, Supplementary Material online). Hyphae of *C. rosea* were frequently observed intermingled, attached and coiled around root hairs, and hyphal swellings putatively representing penetration structures were repeatedly observed (supplementary file S9, Supplementary Material online), although no disease symptoms were observed. In the presence of *F. oxysporum* f. sp. *radicis lycopersici* both fungi were observed on the root surface, and close contact of hyphae indicating mycoparasitism was present at several sites. A potential for mycoparasitism was further suggested by in vitro interactions where hyphae of *C. rosea* IK726 were found to tightly coil around hyphae of *F. oxysporum* f. sp. *radicis lycopersici* (supplementary file S9, Supplementary Material online).

## Discussion

Our phylogenomic analysis shows that the Bionectriaceae family (represented by *C. rosea*) is a sister taxon with the family Nectriaceae (*Fusarium* spp.), with Hypocreaceae (*Trichoderma* spp.) in a basal position, thereby supporting the results from James et al. (2006). Our data also support the close relationship between *Trichoderma* sections Longibrachiatum (*T. longibrachiatum* and *T. reesei*) and Pachybasium (*T. virens*) (Kubicek et al. 2011). Mycoparasitism was previously shown to be the ancestral life style of *Trichoderma* spp. (Druzhinina et al. 2011; Kubicek et al. 2011), but the mycoparasitic lifestyle within Hypocreales may be even older. In fact, certain fossils from the Early Devonian (400 Myr) Rhynie chert sediments depict coiling hyphae surrounding partially degraded perithecia, which are interpreted as remnants of mycoparasites (Hass et al. 1994; Taylor et al. 2005). Whether mycoparasitism in *C. rosea* represents an ancestral trait shared by *Clonostachys* spp. and *Trichoderma* spp., or represents a case of convergent evolution, requires better understanding of the mycoparasitic potential of species within Bionectriaceae.

We hypothesized that the mycoparasitic lifestyle in Bionectriaceae and Hypocreaceae results in selection for similar interaction mechanisms. However, the majority of expanded gene families in *C. rosea* did not evolve in the same manner in *Trichoderma* spp., indicating differences in the underlying mechanisms of antagonism. One major difference concerns the ABC transporter gene family, where the lifestyle of *C. rosea* results in selection for increased numbers of genes in groups B-III, G-I, and G-V, presumably reflecting the need for tolerance toward toxic metabolites produced by the mycoparasite itself, the fungal prey, or plants. Subgroup ABC G-I includes several members with characterized roles in drug resistance, such as Pdr5p, Pdr10p, and Pdr15p in *Saccharomyces cerevisiae* (Rogers et al. 2001). Interestingly, the TAABC2 transporter from *T. atroviride* belongs to the ABC G-V group, and is responsible for resistance toward exogenous toxins from other fungi (Ruocco et al. 2009). The ABC transporter genes *abcG5* and *abcG29* (groups G-I and G-V, respectively) from *C. rosea* are induced by ZEN (Kosawang et al. 2014) and deletion of *abcG5* results in reduced ZEN tolerance and partial loss of the ability to protect barley against mycotoxigenic *F. graminearum* (Dubey et al. 2014).

Deletion of the group B-III gene *abc3* in *M. oryzae* results in mutants sensitive to valinomycin and actinomycin D, but highly sensitive to oxidative stress (Sun et al. 2006). The *M. oryzae* Δ*abc3* mutants also contained higher endogenous levels of H_2_O_2_, and if this function can be proven also for *C. rosea* B-III ABC transporters it is interesting to speculate about a connection between the expansions of ABC B-III genes and the AA3_2 subfamily genes encoding H_2_O_2_-producing enzymes. Group C-V ABC transporters are hypothesized to be involved in secretion of endogenous toxins due to their close association with secondary metabolite biosynthesis clusters (von Döhren 2009; Kovalchuk and Driessen 2010). The increased numbers of ABC C-V genes in *T. virens*, but not *C. rosea*, may therefore be connected with the dependence on gliotoxin production in this species (Atanasova, Le Crom, et al. 2013).

The induced expression of several group B and G ABC transporters in *C. rosea* during exposure to ZEN, Boscalid, and bacterial metabolites indicates that membrane transporters may contribute to the high tolerance against these compounds in *C. rosea*. We show that *C. rosea* can tolerate at least 10-fold higher levels of Boscalid than the gray mould pathogen *Botrytis cinerea* (Veloukas et al. 2011), and that *C. rosea* can tolerate toxic compounds produced by different strains of the BCA bacterium *P. chlororaphis* to a substantially higher degree than certain plant pathogenic species such as *Mi.nivale* and *F. oxysporum* f. sp. *radicis lycopersici*. These results may find important agro-industrial application areas, and we show that the combined application of both *C. rosea* and *P. chlororaphis* PCL1391 on tomato seeds protects the emerging seedlings better against root rot caused by *F. oxysporum* f. sp. *radicis lycopersici*, compared with single application of either BCA.

Mycoparasitic *Trichoderma* species contain high numbers of PKS and NRPS genes (Kubicek et al. 2011), and transcriptome data support the view that production of secondary metabolites that poison the fungal prey is important for both *T. virens* and *T. atroviride*, although *T. atroviride* also induces genes involved in hydrolytic degradation of the cell wall of the fungal prey (Atanasova, Le Crom, et al. 2013). In this context, the high numbers of PKS and NRPS genes in the *C. rosea* genome may suggest a prolific capacity of *C. rosea* to produce, hitherto mostly unknown, polyketide natural products and peptide metabolites. The putative NPS9 peptaibol synthetase in *C. rosea* displays a specificity signature that corresponds well with peptaibols isolated from *C. rosea* strain BAFC3874 (Rodriguez et al. 2011), which contribute to the antifungal activity of *C. rosea* culture filtrates toward *Sclerotinia sclerotiorum* (Rodriguez et al. 2011).

However, very few metabolites are in fact isolated from *C. rosea*. Besides the mentioned peptaibols, *Clonostachys* sp. produce some epipolysulfanyldioxopiperazines with nematicidal activity (Dong et al. 2005), polyterpenoid glisoprenins that inhibit appressorium formation of phytopathogenic fungi (Thines et al. 1998), and cyclic peptides with chitinase inhibitor activity (Arai et al. 2000) that may be involved in protecting the fungus from chitinases produced by itself or by the fungal prey. Certain GH18 subgroup C killer toxin-like chitinases are hypothesized to make the cell wall of the fungal prey more permeable to enhance penetration of toxins (Seidl et al. 2005), and the high subgroup C gene numbers in *T. virens* and *T. atroviride* (15 and 9 genes, respectively) are hypothesized to represent an adaptation to the dependence on toxic metabolites during the mycoparasitic attack (Ihrmark et al. 2010; Seidl-Seiboth et al. 2014). Consequently, the low number (two genes) of subgroup C chitinase genes in *C. rosea* suggests that the products of the orphan secondary metabolite assembly lines in *C. rosea* are not primarily involved in chemical attack.

We hypothesize that certain PKSs are involved in biosynthesis of the pink, yellow, orange, and brown pigments frequently observed in *C. rosea* cultures (Schroers et al. 1999) as various chromophores, for example, polyenes or aromatic systems can be produced by reducing or nonreducing PKSs, respectively. Deletion of the *pks4* gene in *T. reesei* results not only in loss of green conidial pigmentation but also in lower resistance toward toxic metabolites produced by *Alternaria alternata*, *Rhizoctonia solani,* and *S. sclerotiorum* (Atanasova, Knox, et al. 2013). Many PKS genes in *T. reesei* are highly expressed during vegetative growth, which suggest more diverse functional roles than merely in biosynthesis of secondary metabolites (Metz et al. 2011). Two PKS genes of the reducing, lovastatin/citrinin-like type are induced in *T. atroviride* during interaction with *R. solani*, but the identity and function of the resulting products are not known (Atanasova, Le Crom, et al. 2013).

In relation to *T. atroviride* and *T. virens*, and even the saprotrophic *T. reesei*, the fungal cell wall degrading capacity of *C. rosea* appears to be modest. The three *Trichoderma* spp. contain high numbers of GH18 chitinases, GH75 chitosanases, and β-1,3-glucanases (GH17, GH55, GH64 and GH81) (Kubicek et al. 2011), whereas the numbers in *C. rosea* are in most cases lower. The mycoparasitic lifestyle of *T. atroviride* and *T. virens* is correlated with selection for high GH18 subgroup B endochitinase gene numbers (Ihrmark et al. 2010; Seidl-Seiboth et al. 2014). It is therefore significant to observe that *C. rosea* only possesses two B group chitinases, from which one is the ortholog to a conserved ascomycete cell wall bound chitinase (*Chi18-18* in *T. reesei*, *chit-1* in *N. crassa*) with a cell wall plasticizing role during growth (Tzelepis et al. 2012). This suggests that cell wall degradation of the fungal prey may not be a prominent biocontrol trait in *C. rosea*. We must recognize, however, that a low number of genes in a particular isozyme gene family does not necessarily imply a low capacity to degrade a particular substrate, but may suggest a narrower substrate range. For example, the genome of the well-known cellulase and hemicellulase producer *T. reesei* contains fewer cellulase and hemicellulase-encoding genes than most species (Martinez et al. 2008). Deletion of the *C. rosea* GH18 chitinase genes *ech37*, *ech42* (also referred to as *chi1* [Gan et al. 2007]), and *ech58* results in lower in vitro antagonistic activity toward *Fusarium culmorum*, but has no effect on its biocontrol ability toward *F. culmorum* on barley or *Alternaria radicina* on carrots (Mamarabadi et al. 2008).

Sequencing of the *C. rosea* genome and establishment of its taxonomic relationship with mycoparasitic *Trichoderma* spp. have provided a unique opportunity to study key similarities and differences between their respective life strategies, which have direct implications for the implementation of biocontrol in agricultural production systems. A unique feature in *C. rosea* is the high numbers of ABC transporters in subgroups predicted to be involved in drug resistance, which may be exploited in agro-industrial applications to reduce the amount of used pesticides.

## Supplementary Material

Supplementary files S1–S9 are available at *Genome Biology and Evolution* online (http://www.gbe.oxfordjournals.org/).

Supplementary Data
