# Supplementary material for: Insights on the Evolution of Mycoparasitism from the Genome of Clonostachys rosea
Source: Genome Biol Evol. 2015 Jan 8;7(2):465–80. doi: 10.1093/gbe/evu292 (PMC4350171; doi:10.1093/gbe/evu292)
Supplement: Supplementary Data [file supp_7_2_465__index.html]

Insights on the Evolution of Mycoparasitism from the Genome of Clonostachys rosea — Insights on the Evolution of Mycoparasitism from the Genome of Clonostachys rosea — Supplementary Data 

# Insights on the Evolution of Mycoparasitism from the Genome of *Clonostachys rosea*

## Supplementary Data

files

**Files in this Data Supplement:**

- Supplementary Data - pdf file
- Supplementary Data - pdf file
- Supplementary Data - pdf file
- Supplementary Data - pdf file
- Supplementary Data - pdf file
- Supplementary Data - xlsx file
- Supplementary Data - xlsx file
- Supplementary Data - xlsx file
- Supplementary Data - xlsx file
