# Supplementary material for: Insights on the Evolution of Mycoparasitism from the Genome of Clonostachys rosea
Source: Genome Biol Evol. 2015 Jan 8;7(2):465–80. doi: 10.1093/gbe/evu292 (PMC4350171; doi:10.1093/gbe/evu292)
Supplement: Supplementary Data [file supp_evu292_Supplementary_file_S1.pdf]

Supplementary file S1: Materials and Methods

Isolate and culture conditions

The *Clonostachys rosea* isolate IK726, originally isolated from barley roots in Denmark and evaluated extensively regarding its biocontrol efficiency (Jensen et al. 2007), was used for genome sequencing. The isolate was grown in potato dextrose broth (PDB, Thermo Scientific Oxoid, UK) for DNA extraction and on solid Vogel's minimal medium with 1% (w/v) sucrose (Vogel 1956) for RNA extraction. DNA was extracted according to established methods (Sambrook and Russel 2001) and RNA was extracted using the RNeasy Plant Mini Kit (Qiagen, Hilden, Germany) according to manufacturer's instructions.

Genome sequencing and assembly

Base coverage of the *C. rosea* genome was generated using Illumina HiSeq paired end sequencing with an insert length of 0.5 kb and read length of 100 bp using standard library preparation kits. This data was complemented with a mate pair library with 4 kb inserts sequenced on the Life Technologies SOLiD instrument with Exact Call Chemistry to generate sequence reads of 61 bp and 53 bp, forward and reverse, respectively. Illumina reads were quality trimmed using Nesoni clip to only contain bases with $Q > 20$ and also cleaned from adaptor sequences (www.vicbioinformatics.com/software.nesoni). The genome was then assembled with ABySS v. 1.3.3 (Simpson et al. 2009) with k-mer values ranging from 35 to 65. The best assembly was chosen to maximise N50. This was found to be at a k-mer length of 41.

Gene annotation

Protein coding genes in the *C. rosea* genome was annotated using MAKER 2 (Holt and Yandell 2011). Before starting the annotations, we ran RepeatModeler to build a species-specific repeat library which was provided to MAKER. We then configured MAKER to use SNAP (Korf 2004), Augustus (Stanke et al. 2006) and GenemarkES (Ter-Hovhannisyan et al. 2008) for *ab initio* gene calls. Two RNA libraries of pure fungal culture were sequenced on the Illumina HiSeq with 2x100 bp paired end reads. The RNASeq data was then *de novo* assembled with Trinity (Grabherr et al. 2011) and then provided to MAKER 2 as expressed

sequence tag evidence. The evidence set was also complemented with the predicted proteomes of *Trichoderma virens*, *T. atroviride*, *T. reseei* and *Neurospora crassa*. Augustus was trained using the CEGMA gene set and autoAugustus training method while Genemark was self trained using the provided training scripts. SNAP was trained with an iterative procedure as suggested by the authors of MAKER. Initially it was trained with the CEGMA gene annotations. MAKER was then run with only SNAP enabled, to produce a preliminary set of evidence supported SNAP predictions. These were then used to retrain SNAP. When all predictors were trained, MAKER was run on the genome. All gene models were provided with a putative description based on similarity to the Uniprot database based on the best BLAST hit with an expect value less than 1×10^{-20}.

Species phylogeny

The program Composition Vector Tree (CVTree) version 4.2.1 (Xu and Hao 2009) was used for construction of a phylogenomic tree by using whole predicted proteome sequences. Ten species were included for the topology construction, including *C. rosea* (isolate IK726), *Fusarium graminearum* (isolate PH-1), *F. oxysporum* forma specialis (f. sp.) *lycopersici* (isolate FOL 4287), *F. solani* (isolate 77-13-4), *F. verticillioides* (isolate 7600), *T. atroviride* (isolate IMI 206040), *T. reesei* (isolate QM6a), *T. virens* (isolate Gv29-8), *N. crassa* (isolate OR74A) and *Magnaporthe oryzae* (isolate 70-15). Except for *C. rosea*, the predicted proteome sequences of the included species were obtained from the Comparative Fungal Genomics Platform (Choi et al. 2013). In the CVTree method, the proteome of each species was converted into a composition vector based on frequency of short peptides defined by K-tuple length. The K-tuple length was set to seven, which is known to be optimal for fungi (Wang et al. 2009; Zuo et al. 2010). As the composition vector method uses the frequency vector of K amino acids, statistical resampling cannot be done by random replacement of nucleotide or protein sequences. To perform a bootstrap test, we resampled a pool of the whole proteome for each species by randomly choosing a certain amount of sequences. The proportion of resampling for each proteome was determined as 0.6321, as described in the previous study (Zuo et al. 2010). The number of protein sequences was rounded off to the nearest tenth to make it integer. One hundred times of resampling for each proteome and construction of phylogenomic tree were performed. The distance matrices generated by CVTree were converted into neighbour-joining trees by using “Neighbor” in the PHYLIP v.

3.6 package (Felsenstein 2005). A consensus tree of the resulting 100 CVTrees was calculated by using MEGA5 (Tamura et al. 2011) to evaluate bootstrap support of the topology.

Gene family evolution

Branch lengths of the species topology generated by CVTree was calculated with PAML v. 4.4 (Yang 1997, 2007), based on a five-gene alignment including actin, calmodulin, glyceraldehyde 3-phosphate dehydrogenase, DNA-directed RNA polymerase II subunit B and translation elongation factor 1 alpha. Coding gene sequences were retrieved from the respective genome sequences. Each gene was aligned individually using Clustal W (Larkin et al. 2007), and all alignments were then concatenated. The resulting alignment, containing all five genes, was used to calculate branch lengths using codeml in the PAML package (using a global model for dN/dS). The resulting species phylogeny was calibrated to the fossil record by setting the base of the Sordariomycetes to 335 MY ago, according to the fossil recalibration by Lücking et al. (Lücking et al. 2009).

For manually annotated gene families, the predicted proteomes from individual fungal genome sequences were screened using BLASTP (Altschul et al. 1997) in an iterative process, using reference proteins that covered the diversity of the respective gene family as indicated below. For identification of polyketide synthases (PKS) and non-ribosomal peptide synthetases (NRPS), the keto synthase (KS) domain of the aflatoxin PKS, AflC (GenBank: AAS66004.1) of *Aspergillus parasiticus*, and the adenylation (A) domain (A2) of the gliotoxin NRPS GliP (GenBank: EDP52461.1) of *A. fumigatus* were used as query sequences. For ATP-binding cassette (ABC) transporters, *F. graminearum* GenBank: XP_387692, GenBank: ESU10461, GenBank: XP_387341, GenBank: ESU16346 were used as query sequences. For major facilitator superfamily (MFS) transporters, *N. crassa* GenBank: P11636, *Candida albicans* GenBank: P28873, *Saccharomyces cerevisiae* GenBank: Z49210, *Escherichia coli* GenBank: P09836, *E. coli* GenBank: P17448, *E. coli* GenBank: P11551, *E. coli* GenBank: P10903, *N. crassa* GenBank: L36127, *E. coli* GenBank: U40075, *E. coli* GenBank: P41036, *Homo sapiens* GenBank: A55568, *S. cerevisiae* GenBank: P15365, *Pseudomonas putida* GenBank: U10895, *S. cerevisiae* GenBank: P36173 and *E. coli* GenBank: P17583 were used as query sequences. For hydrophobins, *A. fumigatus* GenBank: EAL91643.1, *Schizophyllum commune* GenBank: AAA96324, *N. crassa*

GenBank: AAB24462, *T. reesei* GenBank: CAA92208, *T. reesei* GenBank: P79073, *Cryphonectria parasitica* GenBank: AAA19638, *T. atroviride* GenBank: EHK42285, *T. atroviride* GenBank: EHK42284 and *T. virens* GenBank: EHK16816 were used as query sequences.

For terpenoid synthases (TS), the characterized *Coprinopsis cinerea* GenBank: XP_001832573, GenBank: XP_001836556, GenBank: A8NE23, GenBank: A8NU13, GenBank: XP_001834007, *Botrytis cinerea* GenBank: AAQ16575, *Penicillium roqueforti* GenBank: Q03471 and *Diaporthe amygdali* GenBank: A2PZA5 proteins were used as query sequences. In addition, the *C. rosea* proteome were scanned for TS related domains assigned by InterProScan analysis (Jones et al. 2014). The predicted TS related *C. rosea* candidate proteins were further analysed using InterPro v. 46.0 (Hunter et al. 2012) and Pfam v. 27.0 software (see Supplementary file S5, Table 4 for details).

For glycoside hydrolase family (GH) 18 chitinases, *T. reesei* GenBank: EGR50403, GenBank: DAA05849, GenBank: DAA05860, GenBank: DAA05864, GenBank: DAA05863, GenBank: EGR49542 were used as query sequences. A published pipeline (Moktali et al. 2012; Park et al. 2008) that is based on InterPro domain profiles was used to identify genes encoding cytochrome P450s. Carbohydrate-active enzyme (CAZy) families, other than GH18s, were identified using the CAZy pipeline (Cantarel et al. 2009) as described in Floudas et al. (Floudas et al. 2012).

OrthoMCL v. 2.0.8 (Li et al. 2003) was used to cluster protein sequences from the included fungal proteomes. Gene family evolution analysis was performed on families that contained ≥ 2 genes in at least one species, and were present in ≥ 2 species. The program CAFE (Computational Analysis of gene Family Evolution) v. 3 (Han et al. 2013) was used to test whether gene family sizes were compatible with a stochastic birth and death model, to estimate gene family size in extinct species and to identify lineages with accelerated rates of gene gain or loss. Mutation rate (λ) was estimated from the data and was 0.0024. A separate analysis on ABC transporter subgroups were performed, and included data retrieved from *T. hamatum* (Studholme et al. 2013), *T. longibrachiatum* (Xie et al. 2014) and Kovalchuk and Driessen (2010). Branch lengths of the species topology were based on a five-gene alignment as described above, and λ was 0.001.

Phylogenetic analysis

ABC transporter amino acid sequences were aligned using Clustal W (Larkin et al. 2007) and phylogenetic analyses performed using Neighbour-Joining implemented in MEGA6 (Tamura et al. 2013). The JTT amino acid substitution model (Jones et al. 1992) was used with uniform rates among sites, and pairwise deletion of gaps. Statistical support for phylogenetic grouping was assessed by 100 bootstrap re-samplings.

Analysis of predicted PKS and NRPS proteins.

KS domains or A domains from fungi and bacteria (Table 1) were aligned using the muscle algorithm implemented in MEGA5 (Tamura et al. 2011). Phylogenetic network construction was done with the Splitstree v. 4 program (Huson 1998) using the built-in neighbor-net algorithm. Extraction of A domain specificity signatures was performed by NRPS Predictor2 (Röttig et al. 2011) or manually according to Stachelhaus et al. (Stachelhaus et al. 1999).

Table 1. Genbank accession numbers of PKS synthase genes from ascomycetes and bacteria used for phylogenetic analysis.

**Organism**	**PKS**	**Genbank accession**	**Type**
Acremonium strictum	MOS	CAN87161.2	HR
Aspergillus flavus	PksA	AAS90093.1	NR
Aspergillus fumigatus	Alb1	AAC39471.1	NR
Aspergillus nidulans	PksST	AAA81586.1	NR
Aspergillus nidulans	wA	CAA46695.2	NR
Aspergillus nidulans		AN0150.2	NR
Aspergillus nidulans	ApdA	AN8412.2	Hybrid
Aspergillus nidulans	AptA	XP_663604.1	NR
Aspergillus nidulans	DMOAS	AN8383.2	NR

Aspergillus nidulans	OrsA	EAA59563.1	NR
Aspergillus ochraceoroseus	AflC	ACH72912.1	NR
Aspergillus oryzae	PKSA	BAE71314.1	NR
Aspergillus parasiticus	AflC	AAS66004.1	NR
Aspergillus parasiticus	6-MSAS	AAC23536	PR
Aspergillus terreus	ACAS	XP_001217072.1	NR
Aspergillus terreus	AT4	BAB88689.1	NR
Aspergillus terreus	ATEG_00325	XP_001210411.1	Hybrid
Aspergillus terreus	ATEG_09961	XP_001209263	Hybrid
Aspergillus terreus	LovB	AAD39830.1	HR
Aspergillus terreus	LovF	AAD34559.1	HR
Bipolaris oryzae		BAD22832.1	NR
Byssochlamys nivea	6-MSAS	AAK48943	PR
Ceratocystis resinifera	PKSI	AAO60166.1	NR
Cercospora nicotianae	CTB1	AAT69682.1	NR
Chaetomium chiversii	RADS2	ACM42403.1	NR
Colletotrichum lagenarium	PKS1	BAA18956.1	NR
Dothistroma septosporum	PKSA	AAZ95017.1	NR
Elsinoe fawcettii	PKS1	ABU63483.1	NR
Exophiala lecanii-corni	ElPKS1	AAN75188.1	NR
Gibberella fujikuroi	PKS 4	CAB92399.1	NR
Gibberella moniliformis	Fum1p	AAD43562.2	HR
Gibberella moniliformis	FusA	AAT28740.1	Hybrid
Gibberella zeae	Pks4	ABB90283.1	HR
Gibberella zeae	PKS12	AAU10633.1	NR

Gibberella zeae	Pks13	ABB90282.1	NR
Glarea lozoyensis	PKS1	AAN59953.1	NR
Hypomyces subiculosus	Hpm3	ACD39762.1	NR
Metacordyceps chlamydosporia	RDC1	ACD39770.1	NR
Microcystis aeruginosa	McyE	NC_010296	Bacterial
Micromonospora echinospora	CalO5	AAM70355.1	Bacterial
Monascus purpureus	PksCT	BAD44749.1	NR
Nodulisporium sp. ATCC74245	PKS1	AAD38786.1	NR
Ophiostoma piceae	PKSA	ABD47522.2	NR
Penicillium griseofulvum	6-MSAS	ADF47133.1	PR
Penicillium expansum	CheA	CAO91861.1	Hybrid
Saccharopolyspora erythraea	EryAI	AAV51820.1	Bacterial
Sordaria macrospora	PKS	CAM35471.1	NR
Streptomyces antibioticus	ChlB1	AAZ77673.1	Bacterial
Streptomyces viridochromogenes	AviM	AAK83194.1	Bacterial
Wangiella dermatitidis	WdPKS1	AAD31436.3	NR

Abbreviations: HR=highly reducing, NR=nonreducing.

Assessment of fungicide and metabolite tolerance, and biocontrol efficiency

The *in vitro* effect of the fungicide Boscalid (BASF Hellas, Greece) on mycelial growth of *C. rosea* on PDA (Thermo Scientific Oxoid, UK) was studied in triplicates in order to assess half maximal effective concentration (EC_{50}). In dual culture assays, *Pseudomonas chlororaphis* strain MA342 (or sterile water in controls) was streaked at a 6 cm distance from an agar plug of *C. rosea* or *Microdochium nivale* (isolate MG1) in a 9 cm diameter PDA or vegetative peptone agar (VPA, Thermo Scientific Oxoid, UK) plate. Fungal growth was measured in triplicates as the distance between the inoculation point to the hyphal front after

eight days. Percentage fungal growth rate reduction by secreted *P. chlororaphis* metabolites was calculated as: (1-(mean growth in *P. chlororaphis* plates / mean growth in control plates))×100.

Phenazine-1-carboxamide (PCN) was isolated from *P. chlororaphis* strain PCL1391 using Thin Layer Chromatography (TLC) as described previously (Keen et al. 1971). The TLC plate was covered with a thin layer of PDA containing 1×10^6 *C. rosea* or *F. oxysporum* f. sp. *radicis lycopersici* conidia/ml, and assessment of growth on the PCN-containing area was done in triplicates. Biocontrol efficiency was evaluated using a gnotobiotic sand system (Simons et al. 1996), with minor modifications. Plant nutrient solution (Chin-A-Woeng et al. 1998) previously inoculated with 5×10^2 *F. oxysporum* f. sp. *radicis lycopersici* conidia/ml was mixed with sand to give a final concentration of 50 conidia/g sand. Application of BCAs was done by dipping surface-sterilized tomato pre-germinated seeds in a suspension of 1×10^7 *P. chlororaphis* strain PCL1391 cells/ml, 1×10^6 *C. rosea* conidia/ml, or both BCAs mixed together, prior to sowing into the sand. Ten days after sowing, assessment of disease severity was carried out in 20 replicates according to the following disease index scale: 1 = no visible symptoms, 2 = mild symptoms on roots, 3 = severe symptoms on roots and wilting, 4 = dead plants. Data was analysed statistically by analysis of variance (ANOVA) and compared using the Duncan test at $P = 0.05$, using the program SPSS version 16 (SPSS, IL).

Colonization of tomato roots by a green fluorescent protein (*gfp*)-expressing *C. rosea* IK726 isolate (Lübeck et al. 2002) was monitored using a Nikon D-Eclipse C1 confocal microscope, using the default filter set. Digital images were acquired with the manufacturer's software. *F. oxysporum* f. sp. *radicis lycopersici* was transformed to express red fluorescent protein (*rfp*) as described previously (Pantelides et al. 2009), and interactions with *gfp*-expressing *C. rosea* was studied *in vitro* (Bolwerk et al. 2003) and *in planta*.

Gene expression analysis

Gene expression of 11 ABC transporters (Supplementary file S2) was measured using quantitative reverse transcription PCR (qRT-PCR). Two *Fusarium* mycotoxins deoxynivalenol (DON) and zearalenone (ZEN), the fungicide Boscalid and a 2 days post inoculation (dpi) culture filtrate of *P. chlororaphis* strain MA342 were used as exogenous toxic substances to generate environmental stresses with relevance for biocontrol conditions.

C. rosea strain IK726 was cultured in 20 ml liquid Czapek-Dox media (Sigma-Aldrich, MO) in 250 ml Erlenmeyer flasks. After 3 dpi at 25°C, the fungus was subjected to treatment with DON, ZEN or Boscalid at a final concentration of 200 ppm. In the control treatment, DON and ZEN were replaced with an equal volume of methanol while Boscalid was replaced by water. For the *P. chlororaphis* culture filtrate treatment, *C. rosea* was cultured in 100 ml PDB for 7 dpi, harvested by filtration and placed in 100 ml *P. chlororaphis* culture filtrate or fresh VPB as control treatment. Fungal mycelia were subsequently collected after 2 h incubation by vacuum filtration. The harvested mycelia were frozen with liquid nitrogen and kept at -80°C.

Total RNA was extracted from mycelia using RNeasy Plant Mini kit (Qiagen, Germany) according to the manufacturer's protocol. To remove DNA impurities, treatment of DNase I and Ribolock® RNAase inhibitor (Fermentas, Germany) were performed prior to cDNA synthesis using iScript cDNA synthesis kit (Bio-Rad, CA) following the manufacturer's instructions. qRT-PCR was carried out as described previously (Kosawang et al. 2014), except that each treatment was measured in a minimum of three biological replicates, each based on two technical replicates. Gene expression data was analysed by Student's t-test with 95% confidential interval implemented in Statistica v.10 (StatSoft, OK).

References

Altschul SF, et al. 1997. Gapped BLAST and PSI-BLAST: A new generation of protein database search programs. Nucleic Acids Res. 25:3389-3402.

Bolwerk A, et al. 2003. Interactions in the tomato rhizosphere of two *Pseudomonas* biocontrol strains with the phytopathogenic fungus *Fusarium oxysporum* f. sp. *radicis lycopersici*. Mol Plant Microbe In. 16:983-993.

Cantarel BL, et al. 2009. The carbohydrate-active enzymes database (CAZy): An expert resource for glycogenomics. Nucleic Acids Res. 37:D233-D238.

Chin-A-Woeng TFC, et al. 1998. Biocontrol by phenazine-1-carboxamide-producing *Pseudomonas chlororaphis* PCL1391 of tomato root rot caused by *Fusarium radicis lycopersici*. Mol Plant Microbe In. 11:1069-1077.

Choi J, et al. 2013. CFGP 2.0: A versatile web-based platform for supporting comparative and evolutionary genomics of fungi and oomycetes. Nucleic Acids Res. 41:D714-D719.

Felsenstein J. 2005. PHYLIP (phylogeny inference package) version 3.6. Seattle, US:Department of Genome Sciences, University of Washington.
Floudas D, et al. 2012. The paleozoic origin of enzymatic lignin decomposition reconstructed from 31 fungal genomes. Science. 336:1715-1719.
Grabherr MG, et al. 2011. Full-length transcriptome assembly from RNA-seq data without a reference genome. Nat Biotechnol. 29:644-U130.
Han MV, Thomas GWC, Lugo-Martinez J, Hahn MW. 2013. Estimating gene gain and loss rates in the presence of error in genome assembly and annotation using CAFE 3. Mol Biol Evol. 30:1987-1997.
Holt C, Yandell M. 2011. MAKER2: An annotation pipeline and genome-database management tool for second-generation genome projects. BMC Bioinformatics. 12:491.
Hunter S, et al. 2012. InterPro in 2011: New developments in the family and domain prediction database. Nucleic Acids Res. 40:D306-D312.
Huson DH. 1998. Splitstree: Analyzing and visualizing evolutionary data. Bioinformatics. 14:68-73.
Jensen DF, et al. 2007. Development of a biocontrol agent for plant disease control with special emphasis on the near commercial fungal antagonist *Conostachys rosea* strain 'IK726'. Australas Plant Path. 36:95-101.
Jones P, et al. 2014. InterProScan 5: Genome-scale protein function classification. Bioinformatics. 30:1236-1240.
Jones DT, Taylor WR, Thornton JM. 1992. The rapid generation of mutation data matrices from protein sequences. Comput Appl Biosci. 8:275-282.
Keen NT, Sims JJ, Erwin DC, Rice E, Partridge J. 1971. 6a-hydroxyphaseollin: Antifungal chemical induced in soybean hypocotyls by *Phytophthora megasperma* var. *sojae*. Phytopathology. 61:1084-1089.
Korf I. 2004. Gene finding in novel genomes. BMC Bioinformatics. 5:59.
Kovalchuk A, Driessen AJM. 2010. Phylogenetic analysis of fungal ABC transporters. BMC Genomics. 11:177.
Larkin MA, et al. 2007. Clustal W and clustal X version 2.0. Bioinformatics. 23:2947-2948.
Li L, Stoeckert CJ, Roos DS. 2003. OrthoMCL: Identification of ortholog groups for eukaryotic genomes. Genome Res. 13:2178-2189.
Lübeck M, et al. 2002. GUS and GFP transformation of the biocontrol strain *Clonostachys rosea* IK726 and the use of these marker genes in ecological studies. Mycol Res. 106:815-826.

Lücking R, Huhndorf S, Pfister DH, Plata ER, Lumbsch HT. 2009. Fungi evolved right on track. Mycologia. 101:810-822.
Moktali V, et al. 2012. Systematic and searchable classification of cytochrome p450 proteins encoded by fungal and oomycete genomes. BMC Genomics. 13:525.
Pantelides IS, Tjamos SE, Striglis IA, Chatzipavlidis I, Paplomatas EJ. 2009. Mode of action of a non-pathogenic *Fusarium oxysporum* strain against *Verticillium dahliae* using real time qPCR analysis and biomarker transformation. Biol Cont. 50:30-36.
Park J, et al. 2008. Fungal cytochrome p450 database. BMC Genomics. 9:402.
Röttig M, et al. 2011. NRPSpredictor2-a web server for predicting NRPS adenylation domain specificity. Nucleic Acids Res. 39:W362-W367.
Sambrook J, Russel D. 2001. Molecular cloning: A laboratory manual. New York, US:Cold Spring Harbor Laboratory Press.
Simons M, et al. 1996. Gnotobiotic system for studying rhizosphere colonization by plant growth-promoting *Pseudomonas* bacteria. Mol Plant Microbe In. 9:600-607.
Simpson JT, et al. 2009. ABySS: A parallel assembler for short read sequence data. Genome Res. 19:1117-1123.
Stachelhaus T, Mootz HD, Marahiel MA. 1999. The specificity-conferring code of adenylation domains in nonribosomal peptide synthetases. Chem Biol. 6:493-505.
Stanke M, Schoffmann O, Morgenstern B, Waack S. 2006. Gene prediction in eukaryotes with a generalized hidden markov model that uses hints from external sources. BMC Bioinformatics. 7:62.
Studholme DJ, et al. 2013. Investigating the beneficial traits of *Trichoderma hamatum* GD12 for sustainable agriculture – insights from genomics. Front Plant Sci. 4:258.
Tamura K, et al. 2011. MEGA5: Molecular evolutionary genetics analysis using maximum likelihood, evolutionary distance, and maximum parsimony methods. Mol Biol Evol. 28:2731-2739.
Tamura K, Stecher G, Peterson D, Filipski A, Kumar S. 2013. MEGA6: Molecular evolutionary genetics analysis version 6.0. Mol Biol Evol. 30:2725-2729.
Ter-Hovhannisyan V, Lomsadze A, Chernoff YO, Borodovsky M. 2008. Gene prediction in novel fungal genomes using an *ab initio* algorithm with unsupervised training. Genome Res. 18:1979-1990.
Vogel HJ. 1956. A convenient growth medium for *Neurospora* (medium N). Microbial Genet Bull. 13:42-43.

Wang H, Xu Z, Gao L, Hao BL. 2009. A fungal phylogeny based on 82 complete genomes using the composition vector method. BMC Evol Biol. 9:195.

Xie B, et al. 2014. Comparative genomics provide insights into evolution of *Trichoderma* nutrition style. Genome Biol Evol. 6:379-390.

Xu Z, Hao BL. 2009. CVTree update: A newly designed phylogenetic study platform using composition vectors and whole genomes. Nucleic Acids Res. 37:W174-W178.

Yang ZH. 1997. PAML: A program package for phylogenetic analysis by maximum likelihood. Comput Appl Biosci. 13:555-556.

Yang ZH. 2007. PAML 4: Phylogenetic analysis by maximum likelihood. Mol Biol Evol. 24:1586-1591.

Zuo G, Xu Z, Yu H, Hao B. 2010. Jackknife and bootstrap tests of the composition vector trees. Genomics Proteomics Bioinformatics. 8:262-267.
