# Supplementary material for: Insights on the Evolution of Mycoparasitism from the Genome of Clonostachys rosea
Source: Genome Biol Evol. 2015 Jan 8;7(2):465–80. doi: 10.1093/gbe/evu292 (PMC4350171; doi:10.1093/gbe/evu292)
Supplement: Supplementary Data [file supp_evu292_Supplementary_file_S2.pdf]

Supplementary file S2: ABC transporter genes and oligonucleotides used for quantitative RT-PCR.

Gene name	Forward primer (5´→ 3')	Reverse primer (5´→ 3')	Phylogenetic group[1]
abcB1	GAAGCGCTCATCCCCCACTG	GCGGTTCGGATTTGACGGATAG	ABC B-I
abcB3	GGACAAAGACGCCCACTCG	GGTTCGTCCACTTCGGTTCCT	ABC B-II
abcB4	TCGCGGTCAAGGAGGATACTA	CCAGAGACGGGCGAATGAG	ABC B-II
abcB18	CTGGCCGAGATGACTTGGGTAAAT	AGGTCCGGGCTGAATGTCTGTT	ABC B-IV
abcB20	TCAAAGGAAACCGGGCAGAAT	AGACGGCTTGGACAGGGTTAGAGA	ABC B-IV
abcB26	GATGGCTCGCGATTGCTCTC	AGTAAGGCCGAAAAGTTTGATGTCT	ABC half size B-IV
abcC12	CAACCACGCGACTCACCATTC	ATAACAACGAGGCGGCAAGATAGA	ABC C-V
abcC14	GACCCGAGTATCTGGAGCAAAACA	TCGGGACCCAAGTAGAATGAGC	ABC C-V
abcG8	GCAGTACTTTGAGGAGCTGGGTTTC	GGGCTTCGAGGCGTCTTATTCT	ABC G-I
abcG11	GGTTCCCGACTTCCTCACTTCAA	CGGGGGACCTGGCTCTCG	ABC G-I
abcG25	CAGGCCGAGTCCATATTGTCTTCT	TGCTCCAGGGGCGATTGA	ABC G-V

[1] Nomenclature of phylogenetic classification is according to Kovalchuk and Driessen (2010).

References

Kovalchuk A, Driessen AJM. 2010. Phylogenetic analysis of fungal ABC transporters. BMC Genomics. 11:177.
