# Supplementary material for: Insights on the Evolution of Mycoparasitism from the Genome of Clonostachys rosea
Source: Genome Biol Evol. 2015 Jan 8;7(2):465–80. doi: 10.1093/gbe/evu292 (PMC4350171; doi:10.1093/gbe/evu292)
Supplement: Supplementary Data [file supp_evu292_Supplementary_file_S5.pdf]

Supplementary file S5. Predicted polyketide synthase, non-ribosomal peptide synthetase and terpenoid synthase assembly lines in *Clonostachys rosea*.

Table 1. Predicted polyketide synthases in the *Clonostachys rosea* genome.

**PKS**	**Protein Id**	**Length (aa)**	**Domains**	**Type**
PKS1	BN869_T00000928_1	1787	SAT KS AT PT T	NR
PKS2	BN869_T00010693_1	2622	SAT KS AT PT T MT R	NR
PKS3	BN869_T00000911_1	2417	SAT KS AT PT T MT TE	NR
PKS4	BN869_T00006898_1	2555	SAT KS AT PT T MT R	NR
PKS5	BN869_T00011525_1	2700	KS AT DH MT ER KR T	HR
PKS6	BN869_T00008325_1	2672	KS AT DH MT ER KR T	HR
PKS7	BN869_T00008884_1	2598	KS AT DH MT ER KR T	HR
PKS8	BN869_T00011033_1	2357	KS AT DH ER KR T	HR
PKS9	BN869_T00006567_1	2530	KS AT DH ER KR T	HR
PKS10	BN869_T00013077_1	2375	KS AT DH ER KR T	HR
PKS11	BN869_T00008198_1	2428	KS AT DH ER KR T	HR
PKS12	BN869_T00010694_1	2600	KS AT DH MT ER KR T	HR
PKS13	BN869_T00013279_1	2584	KS AT DH MT ER KR T	HR
PKS14	BN869_T00006415_1	2502	KS AT DH MT ER KR T	HR
PKS15	BN869_T00009642_1	2349	KS AT DH ER KR	HR
PKS16	BN869_T00013307_1	2403	KS AT DH MT ER KR T	HR
PKS17	BN869_T00012010_1	2238	KS AT DH ER KR T	HR
PKS18	BN869_T00007381_1	2505	KS AT DH MT ER KR T	HR
PKS19	BN869_T00013339_1	2346	KS AT DH ER KR T	HR
PKS20	BN869_T00010238_1	2411	KS AT DH MT ER KR T	HR
PKS21	BN869_T00007502_1	2544	KS AT DH MT ER KR T	HR
PKS22	BN869_T00009884_1	2490	KS AT DH MT ER KR T	HR
PKS23	BN869_T00010141_1	2614	KS AT DH MT ER KR T	HR

PKS24	BN869_T00005452_1	2561	KS AT DH MT ER KR T	HR
PKS25	BN869_T00012373_1	2707	KS AT DH MT ER KR T	HR
PKS26	BN869_T00013405_1	2418	KS AT DH MT ER KR	HR
PKS27	BN869_T00009545_1	2000	KS AT DH MT ER	HR
PKS28	BN869_T00005753_1	2345	KS AT DH ER KR T	HR
PKS29	BN869_T00010453_1	2378	KS AT DH MT ER KR T	HR
PKS30	BN869_T00008531_1	271	KS	fragment
PKS31	BN869_T00001420_1	430	KS	fragment
PKS32	BN869_T00013212_1	3889	KS AT DH ER KR T C A T R	hybrid

Abbreviations: AT=acyl transferase, DH=dehydratase, ER=enoyl reductase, KR=ketoreductase, KS=ketosynthase, PT=product template domain (first ring cyclase), R=reducatse, SAT=starter unit acyl transferase, TE=thioesterase HR=highly reducing, NR=nonreducing, hybrid=PKS-NRPS hybrid.

Table 2. Predicted non-ribosomal peptide synthetase gene clusters and their proposed domain structure in the *Clonostachys rosea* genome.

NRPS Gene cluster	Protein ID	Length (aa)	Domains	Proposed function/ comment
NPS1	BN869_T00013362_1	2206	$A_1T_1C_1$ A_2T_2 C_2T_3	putative ETP synthetase
NPS2	BN869_T00008995_1	1022	A_1 X	paralog of BN869_T00008995_1
NPS3 (NPS3A, NPS3B)	BN869_T00006601_1	984	A_1 X	paralog of BN869_T00008995_1
	BN869_T00006619_1	1285	A_1T_2 R	
NPS4 (NPS4A, NPS4B)	BN869_T00007296_1	4331	AT T_0 C_1 $A_1T_1C_1$ $A_2T_2C_2$ $A_3T_3C_3$	putative peptaibol synthetase (11 modules)
	BN869_T00007295_1	8453	$A_4T_4C_4$ $A_5T_5C_5$ $A_6T_6C_6$ $A_7T_7C_7$ $A_8T_8C_8$ $A_9T_9C_9$ $A_{10}T_{10}C_{10}$ $A_{11}T_{11}$R	
NPS5	BN869_T00010383_1	5443	$A_1T_1C_1$ $A_2T_2C_2$ $A_3T_3C_3$ $A_4T_4C_4$ $A_5T_5C_5$	
NPS6	BN869_T00000574_1	9406	$A_1T_1C_1$ $A_2T_2C_2$ $A_3T_3C_3$ $A_4T_4C_4$ $A_5T_5C_5$ $A_6T_6C_6$ $A_7T_7C_7$ $A_8T_8C_8$	
NPS7	BN869_T00012973_1	6047	$A_1T_1C_1$ $A_2T_2C_2$ $A_3T_3C_3$ $A_4T_4C_4$ $A_5T_5C_5$ $A_6T_6C_6$	
NPS8	BN869_T00008264_1	4858	$A_1T_1C_1$ $A_2T_2C_2$ T_3C_3 $A_4T_4C_4$ $T_5C_5T_6C_6$	putative ferricrocin siderophore synthetase
NPS9	BN869_T00013091_1	21815	$A_1T_1C_1$ $A_2T_2C_2$ $A_3T_3C_3$ $A_4T_4C_4$ $A_5T_5C_5$ $A_6T_6C_6$ $A_7T_7C_7$ $A_8T_8C_8$ $A_9T_9C_9$ $A_{10}T_{10}C_{10}$ $A_{11}T_{11}C_{11}$ $A_{12}T_{12}C_{12}$ $A_{13}T_{13}C_{13}$ $A_{14}T_{14}C_{14}$ $A_{15}T_{15}C_{15}$ $A_{16}T_{16}C_{16}$ $A_{17}T_{17}C_{17}$ $A_{18}T_{18}C_{18}$ $A_{19}T_{19}C_{19}$ $A_{20}T_{20}C_{20}$	putative peptaibol synthetase (20 modules)
NPS10	BN869_T00012004_1	3381	$A_1T_1C_1$ A_2T_2 A_3T_3 A_4T_4 A_5T_5 C_5	
NPS11	BN869_T00006571_1	6728	C_0 $A_1T_1C_1$ A_2T_2 A_3T_3 A_4T_4 $A_5T_5C_5$ $A_6T_6C_{6a}$ C_{6b} $A_7T_7C_7$	
NPS12	BN869_T00013096_1	6368	C_0 $A_1T_1C_1$ $A_2T_2C_2$ $A_3T_3C_3$ $A_4T_4C_4$ $A_5T_5C_5$ A_6T_6	putative peptaibol synthetase
NPS13	BN869_T00012005_1	2433	$A_1T_1C_1$ $A_2T_2C_2$	

NPS14	BN869_T00012688_1	1588	T_1C_1 A_2T_2R	Putative incomplete peptaibole synthetase (terminal portion)
NPS15	BN869_T00013464_1	6844	C_0 $A_1T_1C_1$ $A_2T_2C_2$ $A_3T_3C_3$ $A_4T_4C_4$ $A_5T_5C_5$ $A_6T_6C_6$	putative peptaibol synthetase
NPS16	BN869_T00007957_1	12980	C $A_1T_1C_1$ *A_2 T_2C_2 $A_3T_3C_3$ $A_4T_4C_4$ $A_5T_5C_5$ $A_6T_6C_6$ $A_7T_7C_7$ $A_8T_8C_8$ $A_9T_9C_9$ $A_{10}T_{10}C_{10}$ $A_{11}T_{11}C_{11}$ $A_{12}T_{12}C_{12}$	putative peptaibol synthetase
NPS17	BN869_T00006516_1	1178	A_1T_1R	2-aminoadipate reductase

Abbreviations: A=adenylation domain, AT=acyltransferase, C=condensation domain, T=thiolation domain, R=reductase, *=truncated.

Table 3. Adenylation domains of NRPS gene clusters, their non-ribosomal specificity code and predicted substrates. Predictions were performed manually or using the NRPSPredictor2 server.

NRPS Cluster	Protein Id	A-domain	Nonribosomal Code	Predicted substrate (NRPSPredictor2)
NPS1	BN869_T00013362_1	1	DACAIGASMK	Trp#
		2	DVMAYCAILK	Ala
NPS2	BN869_T00008995_1	1	DMWIAACIVK	hydrophobic-aliphatic
NPS3 (NPS3A, NPS3B)	BN869_T00006601_1	1	DMWIAACIVK	hydrophobic-aliphatic
	BN869_T00006619_1	2	--WLWNVEVK	hydrophobic-aliphatic
NPS4 (NPS4A, NPS4B)	BN869_T00007296_1	1	DFCFFAGVVK	Aib/Iva
		2	DLGIVAAVIK	hydrophobic-aliphatic
		3	DAMVIGAVIK	hydrophobic-aliphatic
	BN869_T00007295_1	4	DQLMLGAVIK	hydrophobic-aliphatic
		5	DIGWAAGLVK	Aib/Iva
		6	DLFFGGLSCK	Pro
		7	DLTYSGGVHK	Glu
		8	DFVCLGAVMK	Gly/Ala
		9	DGGMVGGNYK	Gln
		10	DIGFLAH-DK	Ile
		11	DASVNGAISK	Leu
NPS5	BN869_T00010383_1	1	DVSNLSTFKK	Ala
		2	DVIGVAWVAK	hydrophobic-aliphatic
		3	DVVNVGWAAK	Ala
		4	DAWGYGMSTK	Phe,Trp
		5	DVSYAGIQAK	hydrophobic-aliphatic
NPS6	BN869_T00000574_1	1	DVSNIALIGK	Leu
		2	DVCILILTFK	hydrophobic-aliphatic

		3	DTNDIGCPTK	Orn, Lys, Arg
		4	DVSDLSTVKK	hydrophobic-aliphatic
		5	DTQEIGSPTK	Asp,Asn,Glu,Gln,Aad
		6	HIRNIDTMTK#	unknown
		7	DQSDIGCPTK	Leu
		8	DISDVAMVKK	hydrophobic-aliphatic
NPS7	BN869_T00012973_1	1	DVLIIAGMCK	hydrophobic-aromatic
		2	DILIVVGLCK	hydrophobic-aromatic
		3	DLEEVGTPTK	Asp,Asn,Glu,Gln,Aad
		4	DVDEIGTPTK	Asp,Asn,Glu,Gln,Aad
		5	DVDH--TPTK	Ala
		6	DLEEVGTPTK	Asp,Asn,Glu,Gln,Aad
NPS8	BN869_T00008264_1	1	DVFELIMIHK	Gly#
		2	DVFSVAGNIK	Ser#
		3	DVLDIGAIGK	Aho#
NPS9	BN869_T00013091_1	1	DVSMLVAIFK	Ala
		2	DLGYMAACFK	Aib/Iva
		3	DLGFLAGCFK	Aib/Iva
		4	DVGMVTSINK	Aad
		5	DFCFFAAVCK	Aib/Iva
		6	DMGWVAGCLK	Aib/Iva
		7	DVCMMVAIFK	Aib/Iva
		8	DYCGIGAICK	Cys
		9	DFGFLAVFLK	Aib/Iva
		10	DWMFGGFINK	Pro
		11	DFGLQGAYAK	Aib/Iva
		12	DLGYLAGCFK	Aib/Iva
		13	DLGYVAACFK	Aib/Iva
		14	DIIFGGLICK	Pro
		15	DATLIGAVCK	Leu
		16	DFGYVGATIK	Aib/Iva

		17	DLGYLAGCFK	Aib/Iva
		18	DIIFGGLICK	Pro
		19	DFGMFAVFAK	Aib/Iva
		20	DFAFVALVGK	Aib/Iva
NPS10	BN869_T00012004_1	1	DMYFVASPGK	Leu
		2	DFSFVGIVMK	Leu
		3	DMFLAAVPGK	aromatic
		4	DLYQVAVPCK	Pro
		5	DAMLIGAVIK	hydrophobic-aliphatic
NPS11	BN869_T00006571_1	1	DIHIVGAPHK	Leu
		2	DVSENGTPTK	hydrophilic
		3	DDFSLGGCTK	Lys
		4	DAMFLIAPLK	hydrophobic-aliphatic
		5	DVSATGSIQK	hydrophilic
		6	DVYLIVATNK	Cys
		7	DAFVLGAVIK	hydrophobic-aliphatic
NPS12	BN869_T00013096_1	1	DMGYVIACIK	hydrophobic-aliphatic
		2	DMYSVALCCK	Pro
		3	DFGLGGMFAK	Ala/Gly
		4	DFGFIGACFK	Aib/Iva
		5	DIIFGGLICK	Pro
		6	DFGFFAGVAK	Aib/Iva
NPS13	BN869_T00012005_1	1	DICHCSVIVK	hydrophobic-aliphatic
		2	DFSFAGVVIK	hydrophobic-aliphatic
NPS14	BN869_T00012688_1	3	DVYFVCGPFK	hydrophobic-aliphatic
NPS15	BN869_T00013464_1	1	DFGYMGAVFK	Aib/Iva
		2	DLFFGGLSCK	hydrophobic-aliphatic
		3	DFGLLGAFIK	hydrophobic-aliphatic
		4	DMGYAAGVFK	Aib/Iva
		5	DMLFGGINCK	hydrophobic-aliphatic

		6	DFGLGGFFAK	hydrophobic-aliphatic
NPS16	BN869_T00007957_1	1	DFMIFGAVCK	hydrophobic-aliphatic
		2	n.a.	
		3	DLGYLAACLK	Aib/Iva
		4	DIIFGGLICK	Pro
		5	DFACIAIVAK	hydrophobic-aliphatic
		6	DAGMMTGVFK	Aib/Iva
		7	DIIFGGLICK	Pro
		8	DFGFIAGIVK	Aib/Iva
		9	DMGYVAAVLK	Pro
		10	DLGYLAGCFK	Iva
		11	DLGYLAGCFK	Iva
		12	DFAWVGLVAK	hydrophobic-aliphatic
NPS17	BN869_T00006516_1	1	DPRHFVLRAK	Aad
PKS32 (PKS-NRPS hybrid)	BN869_T00013212_1	1	DVSYIAEVQK	hydrophobic-aliphatic

Abbreviations of non-proteinogenic substrates: Aad=2-aminoadipic acid, Aho=N^5-acyl-N^5-hydroxyornithine, Aib=2-aminoisobutyric acid, Iva=isovaline, Orn=ornithine. n.a.=not applicable, #=manually extracted NRPS code.

Table 4. Predicted terpenoid synthases (TS)[1] in the *Clonostachys rosea* genome.

TS	Protein ID	Gene length (bp)	InterProScan/PFAM ID family domains	Description
TES1	BN869_T00002098_1	2283	IPR008949, IPR017446, IPR000092, PF00348, PF03936	Terpene synthase family, metal binding domain
TES2	BN869_T00006434_1	1068	IPR008949, PF03936	Terpene synthase family, metal binding domain
TES3	BN869_T00002452_1	963	IPR008949, PF03936, PF06330	Terpene synthase family, metal binding domain/Trichodiene synthase (TRI5)
TES4	BN869_T00004037_1	1299	IPR008949, IPR017446, IPR000092, PF00348	Polyprenyl synthase
TES5	BN869_T00011314_1	1029	IPR008949, IPR017446, IPR000092, PF00348	Polyprenyl synthase
TES6	BN869_T00002638_1	1098	IPR008949, IPR017446, IPR000092, 2 domains of PF00348	2 domains of polyprenyl synthase
TES7	BN869_T00000651_1	1389	IPR008949, IPR002060, IPR006449, PF00494	Squalene/phytoene synthase
TES8	BN869_T00005085_1	1743	IPR008949, IPR002060,IPR017825, PF00494	Squalene/phytoene synthase and lycopene cyclase

[1] The identification of predicted TS related proteins were based on domain searches using any of the following domains for either InterProScan: IPR008949 = terpenoid_synth, IPR017446 = polyprenyl_synth-rel, IPR000092 = polyprenyl_synth, IPR002060 = Squ/phyt_synthase, IPR006449 = Squal_synth, IPR017825 = Lycopene_cyclase_dom, or from Pfam v. 27.0 (by using E-value ≤ 1.0): PF00348 = polyprenyl synth, PF03936 = Terpene synth C, PF06330 = TRI5, PF00494 = SQS PSY.
