# Supplementary material for: Insights on the Evolution of Mycoparasitism from the Genome of Clonostachys rosea
Source: Genome Biol Evol. 2015 Jan 8;7(2):465–80. doi: 10.1093/gbe/evu292 (PMC4350171; doi:10.1093/gbe/evu292)
Supplement: Supplementary Data [file supp_evu292_Supplementary_file_S8.pdf]

Supplementary file S8:
Phylogenetic relationships of ABC transporter subgroup C among Hypocreales

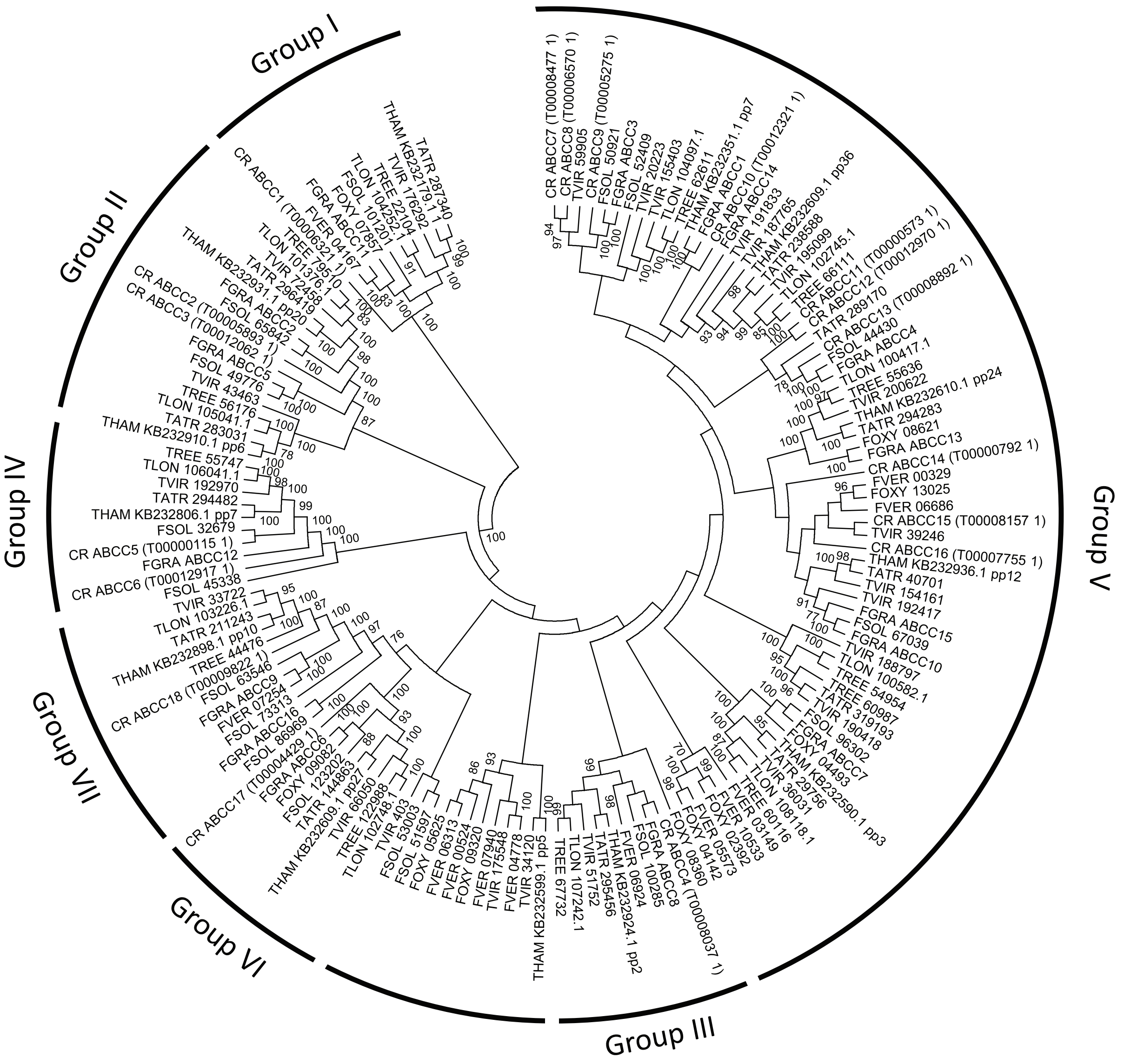

Supplementary file S8:
Phylogenetic relationships of ABC transporter subgroup C among Hypocreales. Predicted amino acid sequences of ABC transporters were aligned by Clustal W and used to construct a phylogenetic tree using the Neighbour-Joining method. ABC C subgroups were designated according to Kovalchuk and Driessen (Kovalchuk and Driessen 2010). Analysis was performed with the MEGA6 software package. Bootstrap support (≥ 70%) values are associated with nodes. Abbreviations: *C. rosea* = CR, *F. graminearum* = FGRA, *F. oxysporum* f. sp. *lycopersici* = FOXY, *F. solani* = FSOL, *F. verticillioides* = FVER, *T. atroviride* = TATR, *T. hamatum* = THAM, *T. longibrachiatum* = TLON, *T. reesei* = TREE, *T. virens* = TVIR.

Reference
Kovalchuk A, Driessen AJM. 2010. Phylogenetic analysis of fungal ABC transporters. BMC Genomics. 11:177.
