# Supplementary material for: Insights on the Evolution of Mycoparasitism from the Genome of Clonostachys rosea
Source: Genome Biol Evol. 2015 Jan 8;7(2):465–80. doi: 10.1093/gbe/evu292 (PMC4350171; doi:10.1093/gbe/evu292)
Supplement: Supplementary Data [file supp_evu292_Supplementary_file_S9.pdf]

Supplementary file S9: Bioassays

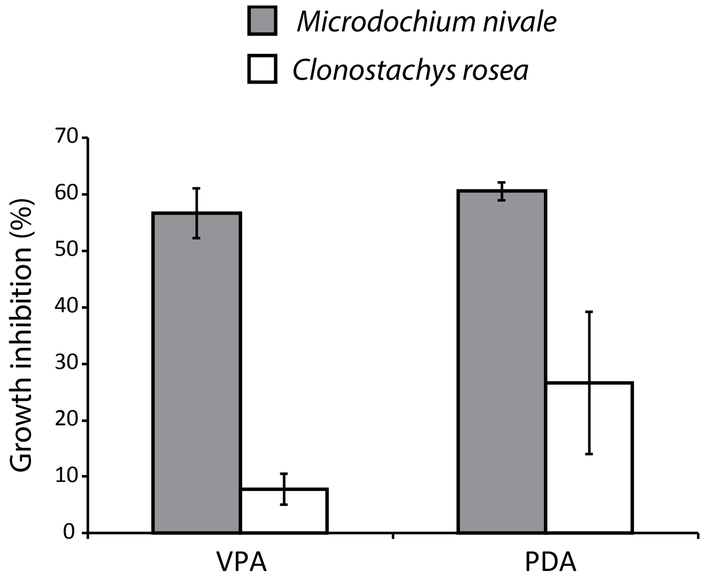

Supplementary file S9: A.
Fungal growth inhibition of *C. rosea* or *M. nivale* by *P. chlororaphis* strain MA342 was assessed in dual culture assays. *P. chlororaphis* MA342 (or sterile water in controls) was streaked at a 6 cm distance from an agar plug of either fungi in a 9 cm diameter PDA or VPA plate. Fungal growth was measured in triplicates as the distance between the inoculation point to the hyphal front after eight days. Percentage fungal growth rate reduction by secreted *P. chlororaphis* metabolites was calculated as: (1-(mean growth in *P. chlororaphis* plates / mean growth in control plates))×100.

Supplementary file S9: Bioassays

++ + ++ -

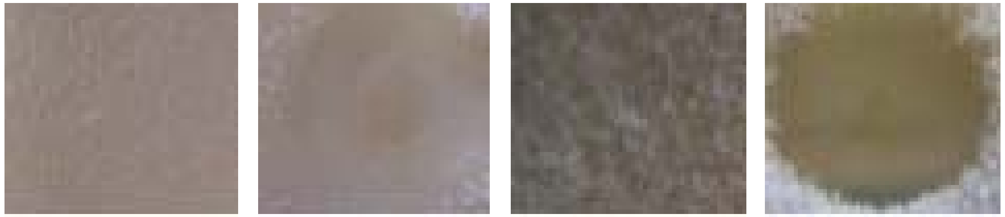

Cros | Cros + PCN | Foxy | Foxy + PCN

Supplementary file S9: B.
Phenazine (PCN) was isolated from *P. chlororaphis* strain PCL1391 using TLC. The TLC plate was covered with a thin layer of PDA containing *C. rosea* (Cros) or *F. oxysporum* f. sp. *radicis lycopersici* (Foxy) conidia, and assessment of growth (- = no growth, + = growth, ++ = vigorous growth) on the PCN-containing area was done in triplicates.

Supplementary file S9: Bioassays

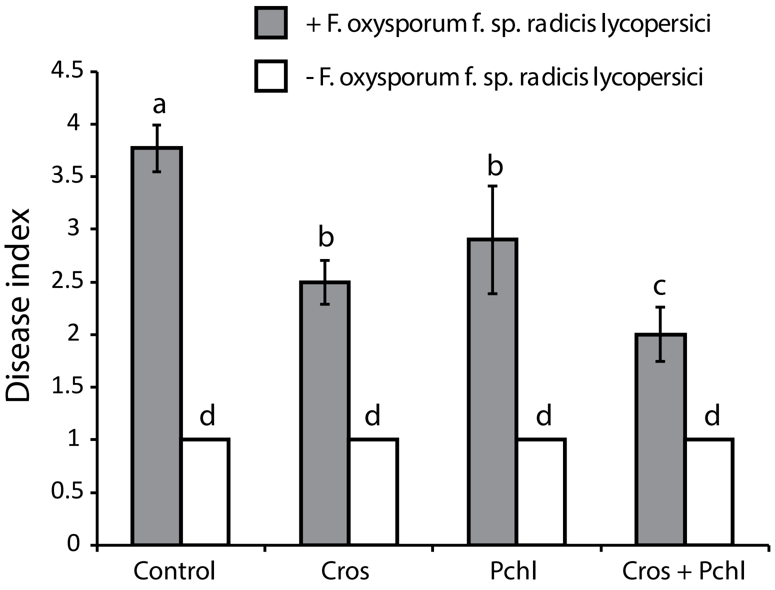

Supplementary file S9: C.
Biocontrol efficiency of *C. rosea*, *P. chlororaphis* strain PCL1391, or the combination, to control Fusarium foot rot disease caused by *F. oxysporum* f. sp. *radicis lycopersici* was evaluated using a gnotobiotic sand system. Pathogen conidia was mixed into the sand, while the application of biocontrol agents (BCAs) was done by dipping surface-sterilized tomato pre-germinated seeds in a suspension of *P. chlororaphis* cells, *C. rosea* conidia, or both BCAs mixed together, prior to sowing into the sand. Ten days after sowing, assessment of disease severity was carried out in 20 replicates according to the following disease index scale: 1 = no visible symptoms, 2 = mild symptoms on roots, 3 = severe symptoms on roots and wilting, 4 = dead plants. Treatments with different letters were significantly different ($P \leq 0.05$) based on the Duncan test.

Supplementary file S9: Bioassays

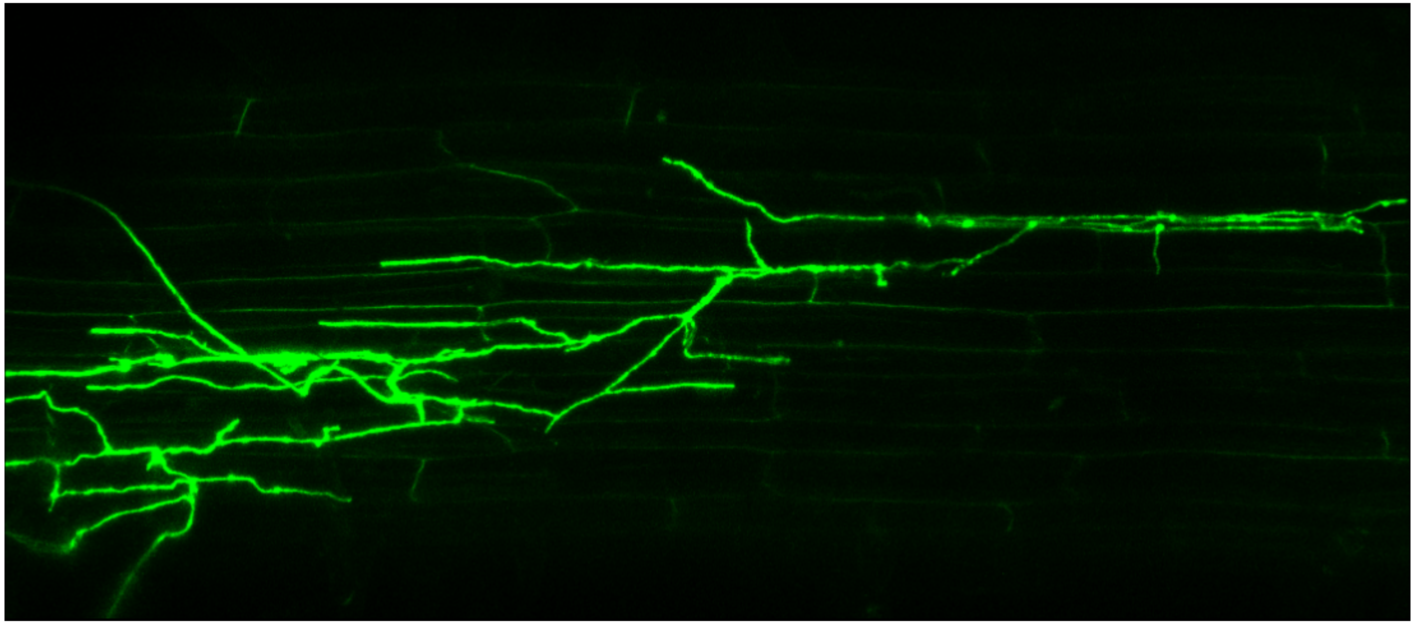

Supplementary file S9: D.
Tomato root colonized by *gfp*-expressing *C. rosea* IK726 6 days after inoculation. Attachment of hyphae on the root surface and growth at the junctions of the epidermal cells.

Supplementary file S9: Bioassays

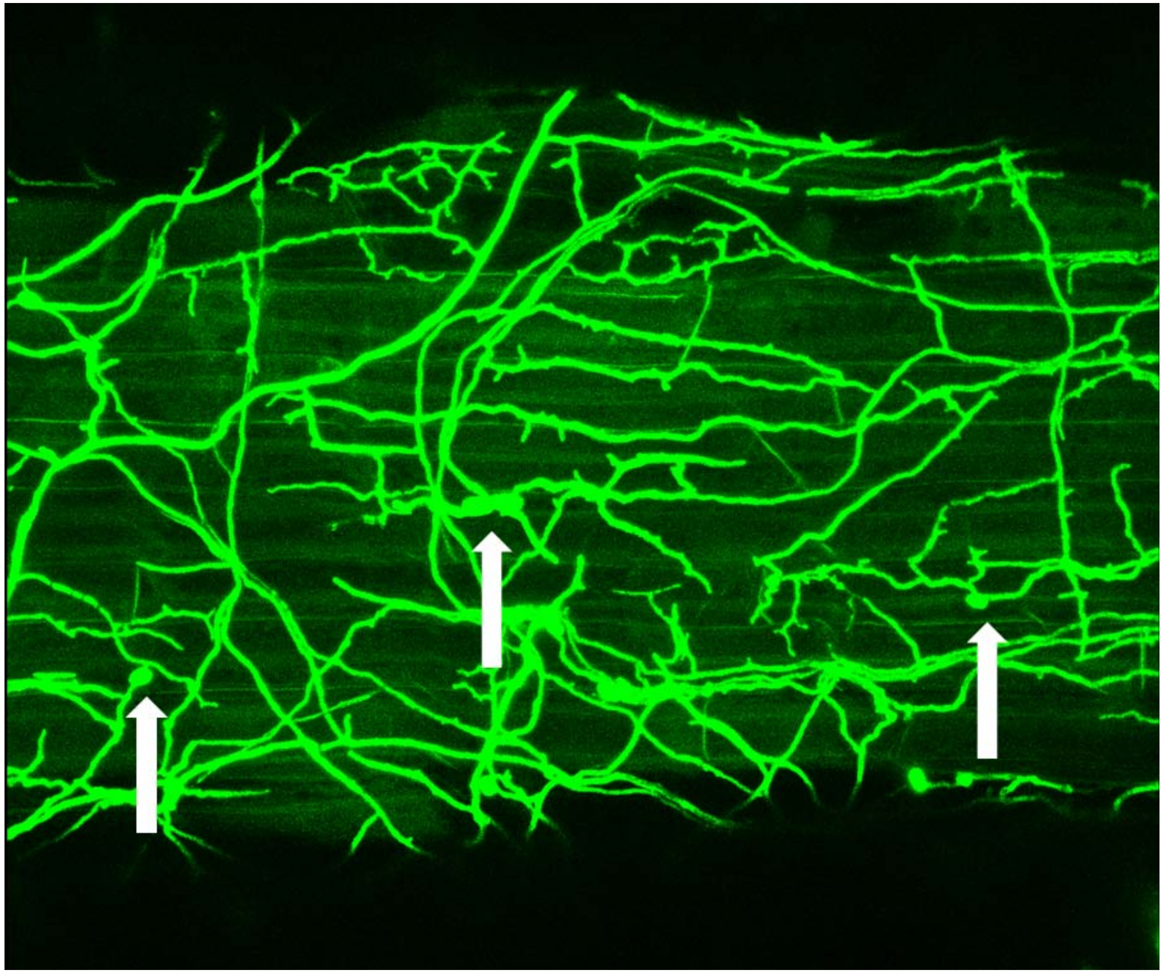

Supplementary file S9: E.
Advanced colonization of tomato root by *gfp*-expressing *C. rosea* IK726 grown along the junctions of the epidermal cells and forming a net of hyphae around the main root 6 days after inoculation. Swellings of hyphae indicate putative penetration points (arrows).

Supplementary file S9: Bioassays

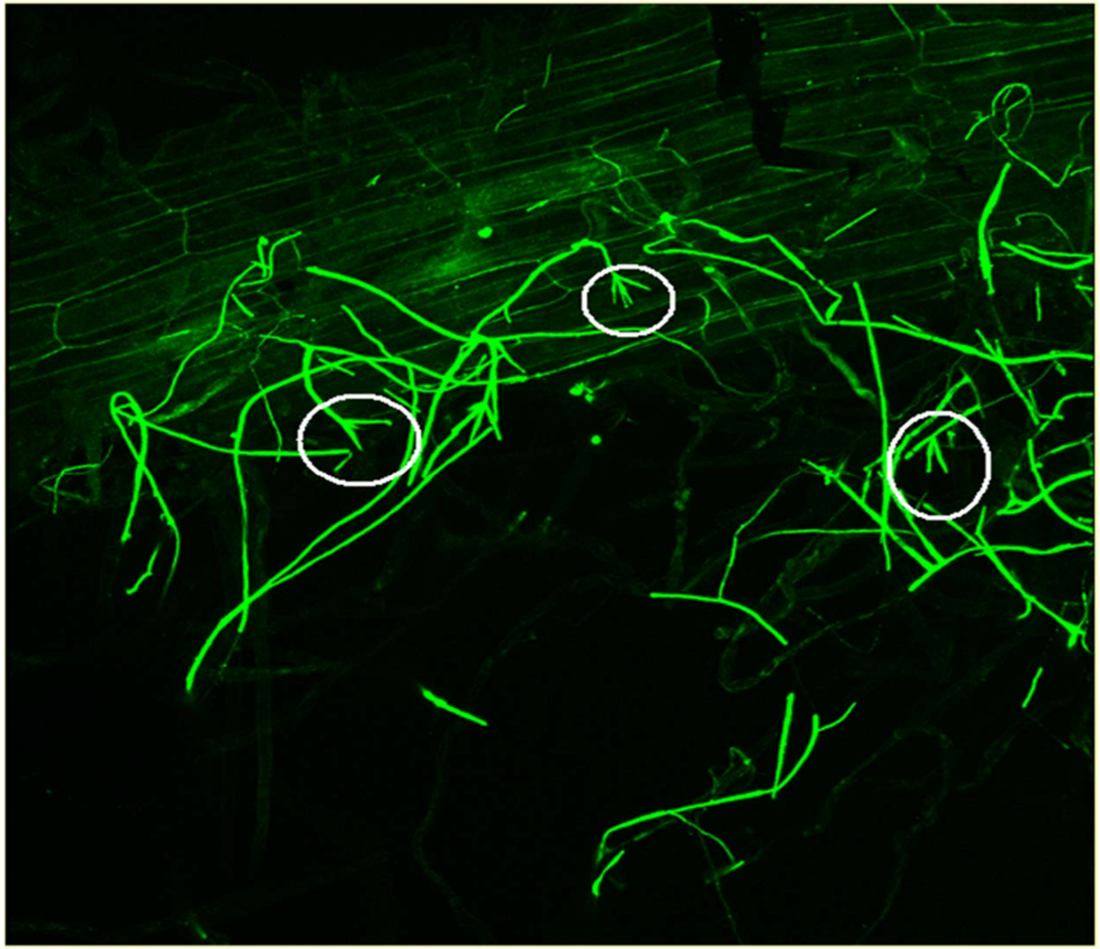

Supplementary file S9: F.
Mingling of *gfp*-expressing *C. rosea* IK726 hyphae with tomato root hairs (right side of picture) and conidiophore appearance (in circles), 6 days after inoculation.

Supplementary file S9: Bioassays

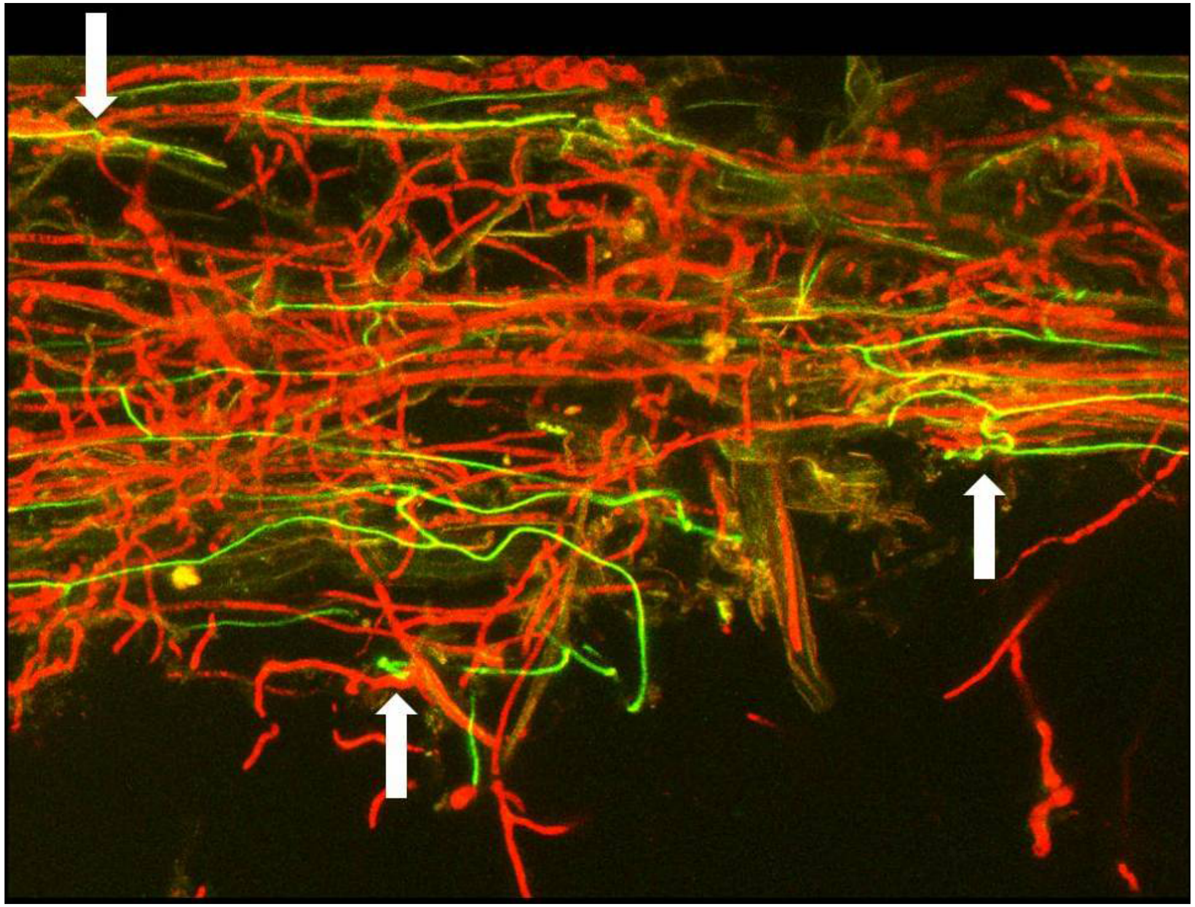

Supplementary file S9: G.
Colonization of tomato root by *gfp*-expressing *C. rosea* IK726 (green) and *rfp*-expressing *F. oxysporum* f. sp. *radicis lycopersici* (red). Close contact of hyphae is observed (arrows).

Supplementary file S9: Bioassays

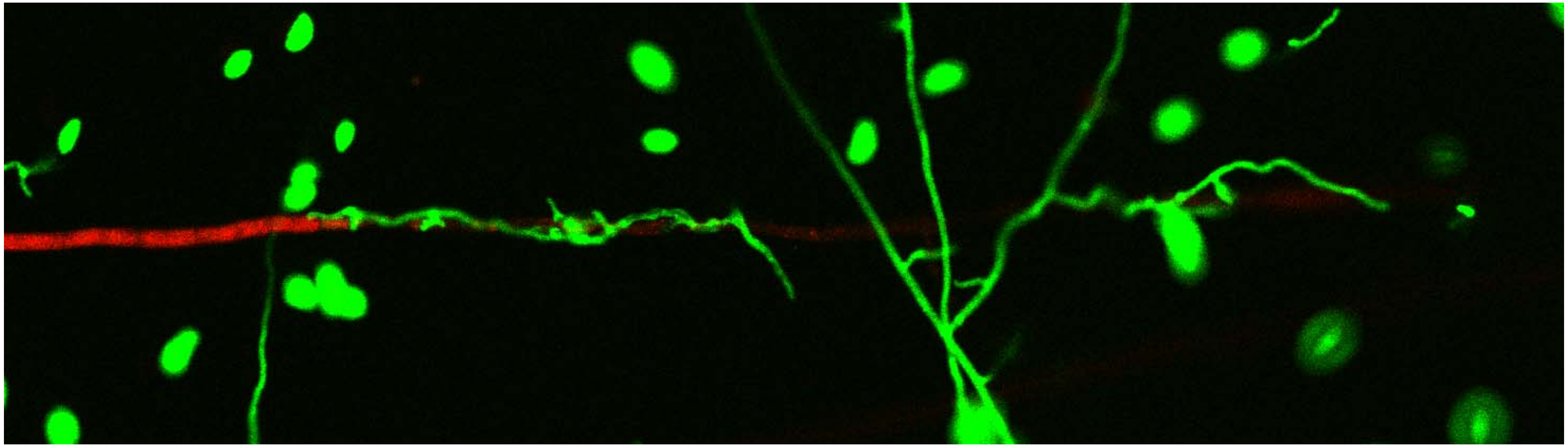

Supplementary file S9: H.
Hyphae of *rfp*-expressing *F. oxysporum* f. sp. *radicis lycopersici* (red) closely coiled by hyphae of *gfp*-expressing *C. rosea* IK726 (green). Spores of *C. rosea* IK726 are visible.
